# Quality of life among women with symptomatic, screen-detected, and interval breast cancer, and for women without breast cancer: a retrospective cross-sectional study from Norway

**DOI:** 10.1007/s11136-021-03017-7

**Published:** 2021-10-26

**Authors:** Nataliia Moshina, Ragnhild S. Falk, Edoardo Botteri, Marthe Larsen, Lars A. Akslen, John A. Cairns, Solveig Hofvind

**Affiliations:** 1 Cancer Registry of Norway, Majorstuen, P.O. 5313, 0304 Oslo, Norway; 2 Oslo Centre for Biostatistics and Epidemiology, Oslo University Hospital, Oslo, Norway; 3 Cancer Registry of Norway, Oslo, Norway; 4 Centre for Cancer Biomarkers CCBIO, Department of Clinical Medicine, Section for Pathology, University of Bergen, Bergen, Norway; 5 Department of Health Services Research and Policy, London School of Hygiene and Tropical Medicine, London, UK; 6 Department of Health and Care Sciences, UiT The Artic University of Norway, P.O. 6050, 9037 Tromsø, Norway; 7 Department of Pathology, Haukeland University Hospital, Bergen, Norway

**Keywords:** Quality of life, Breast cancer screening, Symptomatic breast cancer, Interval breast cancer, EQ-5D-5L

## Abstract

**Purpose:**

Breast cancers detected at screening need less aggressive treatment compared to breast cancers detected due to symptoms. The evidence on the quality of life associated with screen-detected versus symptomatic breast cancer is sparse. This study aimed to compare quality of life among Norwegian women with symptomatic, screen-detected and interval breast cancer, and women without breast cancer and investigate quality adjusted life years (QALYs) for women with breast cancer from the third to 14th year since diagnosis.

**Methods:**

This retrospective cross-sectional study was focused on women aged 50 and older. A self-reported questionnaire including EQ-5D-5L was sent to 11,500 women. Multivariable median regression was used to analyze the association between quality of life score (visual analogue scale 0–100) and detection mode. Health utility values representing women’s health status were extracted from EQ-5D-5L. QALYs were estimated by summing up the health utility values for women stratified by detection mode for each year between the third and the 14th year since breast cancer diagnosis, assuming that all women would survive.

**Results:**

Adjusted regression analyses showed that women with screen-detected (*n* = 1206), interval cancer (*n* = 1005) and those without breast cancer (*n* = 1255) reported a higher median quality of life score using women with symptomatic cancer (*n* = 1021) as reference; 3.7 (95%CI 2.2–5.2), 2.3 (95%CI 0.7–3.8) and 4.8 (95%CI 3.3–6.4), respectively. Women with symptomatic, screen-detected and interval cancer would experience 9.5, 9.6 and 9.5 QALYs, respectively, between the third and the 14th year since diagnosis.

**Conclusion:**

Women with screen-detected or interval breast cancer reported better quality of life compared to women with symptomatic cancer. The findings add benefits of organized mammographic screening.

**Supplementary Information:**

The online version contains supplementary material available at 10.1007/s11136-021-03017-7.

## Plain English summary

1. Why is this study needed?

To our knowledge, this is the first study reporting quality of life outcomes by detection mode, or for women with symptomatic, screen-detected and interval cancer and women without breast cancer.

2. What is the key problem/issue/question this manuscript addresses?

The key questions are if the women without any diagnosis of breast cancer have higher quality of life compared to women with breast cancer regardless of detection mode, and if women with symptomatic cancer have lower quality of life compared to women with screen-detected or interval cancer.

3. What is the main point of your study?

The study aimed to investigate quality of life among women by detection mode, including symptomatic, screen-detected, and interval cancer, and among women without breast cancer.

4. What are your main results and what do they mean?

Women with screen-detected and interval cancer had higher scores of self-reported quality of life compared to women with symptomatic breast cancer in this study. Women with screen-detected breast cancer had higher scores of health utility values obtained from EQ-5D-5L compared to women with symptomatic breast cancer. When compared to women without breast cancer, the quality of life scores for breast cancer survivors were lower. These results are valuable in the policy discussions about cost-effectiveness of mammographic screening and should be considered in favor of organized screening.

## Introduction

Breast cancer is the most common cancer and the second leading cause of cancer death among women in Norway and worldwide [1, 2]. Reduced breast cancer mortality due to early detection and improved treatment has received substantial attention during the last decades [3, 4], while less attention has been given to side effects of the treatment and quality of life [5].

The World Health Organization defines quality of life as an individual’s perception of their position in life related to the culture and value systems in which they live [6]. Health-related quality of life is defined as perceived physical and mental health over time [7]. The concept of quality of life is essential in the evaluation of the side- and long-term effects of cancer treatment. Examples of such effects among breast cancer survivors include cardiac and pulmonary toxicity, reproductive dysfunction, arm lymphedema, neuropathy, skin changes, chronic pain, fatigue, depression and anxiety [8, 9]. Quality-adjusted life years (QALYs) combine the length and the quality of life, and reflect the person’s ability to carry out the activities of daily living without pain and mental disturbance [10]. If the quality of life is measured on a scale where 0 represents ‘death’ and 1 ‘perfect health’, the number of QALYs experienced is estimated by multiplying the expected length of life by the expected quality of life [10].

Organized mammographic screening aims to reduce breast cancer mortality by detecting tumors at an early stage, and thereby reduce the burden of treatment. Results from international review studies and BreastScreen Norway, a population-based cancer screening program, have shown a reduction in breast cancer mortality of 20–30% due to implementation of organized screening [3, 11].

Symptomatic cancer is associated with less favorable prognostic and predictive histopathologic tumor characteristics compared to screen-detected cancer [12]. Women with symptomatic cancer can thus receive more aggressive treatment and are expected to have lower quality of life than those with screen-detected cancer [13]. Although various interventions and quality of life for breast cancer survivors have been evaluated [5, 14], we are not aware of any studies reporting the quality of life or QALYs following treatment of women with symptomatic cancer versus screen-detected cancer.

Interval cancers, breast cancers diagnosed due to symptoms between two screening examinations where the former was negative, might have similar histopathologic tumor characteristics to symptomatic cancers [15]. Women with interval cancer may therefore be treated more aggressively than those with screen-detected cancer, and this might influence their quality of life.

The objective of this study was to compare quality of life among women by detection mode, including symptomatic, screen-detected, and interval cancer, and among women without breast cancer. We also aimed to estimate QALYs among women diagnosed with breast cancer, by detection mode. We hypothesized that women without any diagnosis of breast cancer have higher quality of life compared to women treated for breast cancer regardless of detection mode, and that women with symptomatic cancer would report lower quality of life compared to women with screen-detected cancer due to detection and treatment of the cancer in an earlier stage.

## Materials and methods

### Study design and participants

This retrospective cross-sectional study was based on information from BreastScreen Norway, which offers all female residents aged 50–69 biennial mammographic screening [16]. The program became nationwide in 2005. The annual participation rate in the program is 76% and 84% of the women had attended at least once during the first 20 years of the program [16]. In 2019, 3726 Norwegian women were diagnosed and treated for invasive breast cancer in Norway, including about 1500 screen-detected or interval cancers [2].

A paper-based self-administered questionnaire, developed in collaboration with breast cancer survivors and women without breast cancer, was used to collect data on quality of life and calculate QALYs among women with breast cancer from the third to the 14th year since diagnosis and among women without breast cancer. The Cancer Registry contains screening data since the start-up of the program in 1996 and provided data of the included women for at least 14 years back in time.

The questionnaire included a page with an informed consent regarding privacy and participation in the study. The questionnaire was sent to the women per post between December 11, 2019 and March 14, 2020: women with symptomatic cancer between January 12 and 28, 2020; women with screen-detected cancer between December 18, 2019 and January 10, 2020; women with interval cancer between January 24 and February 20, 2020, and women without breast cancer between February 10 and March 4, 2020. The women were asked to respond to the questionnaire and return it in a prepaid envelope within two weeks. A pilot, including 25 women, was performed four months prior the study start. The overall response rate for the pilot was 44%; 3/6 (50%) women with symptomatic cancer, 4/7 (57%) screen-detected cancer; 3/6 (50%) interval cancer, and 1/6 (17%) women without breast cancer. These response rates were used to estimate the sample size. Power-estimations indicated a need for a minimum of 1000 women in each of the four groups to show a 0.25-point difference in QALY between the groups with 80% power.

A total of 2500 women with symptomatic cancer, 2500 with screen-detected cancer, 2500 with interval cancer and 4000 women without breast cancer were randomly selected from the BreastScreen Norway database. Women aged 50–69 at invitation to screening or at histologically verified invasive breast cancer diagnosis (symptomatic, screen-detected or interval cancer), diagnosed 2006–2017 were eligible for inclusion. Symptomatic cancer was breast cancer diagnosed among women invited to the screening program, but either never attended or last attended more than two years prior to their diagnosis. Screen-detected cancer was breast cancer diagnosed as a result of a positive screening examination. Interval cancer was breast cancer diagnosed 0–24 months after a negative screening examination or 6–24 months after a false positive screening result [16]. Women without breast cancer had no registered invasive breast cancer or ductal carcinoma in situ in the breast in the Cancer Registry database before 2018.

**Fig. 1:**
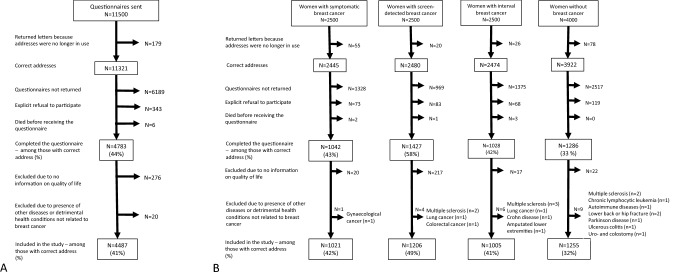
**A** Original study population, exclusions and final study population. **B** Original study population, exclusions and final study population for women with symptomatic, screen-detected and interval cancer, and women without breast cancer

Reporting all cancer types to the Cancer Registry of Norway is mandatory for medical doctors, pathology laboratories and hospitals [17]. Based on data from the Cancer Registry of Norway, we sent the questionnaire solely to women with primary breast cancer. Women without breast cancer were not registered with any cancer type. However, there is a delay in the reporting of cancer cases, which might have resulted in a cancer diagnosis between the date of extraction of the study population and the date when the women received the questionnaire. Women with primary breast cancer might have had other types of cancer diagnosed later in lifetime. Women in all the groups might also have experienced serious health conditions and symptoms significantly decreasing their quality of life, but unrelated to breast cancer. Several symptoms and comorbidities, such as general pain, lymphedema, fatigue, joint pain, heart disease, breathing problems, etc., were included in the questionnaire, and the women, regardless of the study group, were asked to tick the box indicating these symptoms and conditions. The questionnaire also included open fields where women from all the groups were asked to fill in present conditions and comorbidities. The latter information was used to exclude women with other cancer types or serious health conditions influencing quality of life (Fig. 1b). The questionnaire included questions about the treatment and relapse. As women with relapse were included in the study, it is assumed that some of them were in active treatment when they responded to the questionnaire.

The study was approved by the Regional Committees for Medical and Health Research Ethics (N28484) and registered at Clinical.Trials.gov (NCT03877029). A running number for each questionnaire was used to merge the self-reported information with the database. The data were de-identified prior to analyses. Research data used in the analyses will be available by request, according to the General Data Protection Regulation [18].

### Variables and data measurement

The questionnaire collected information about height (cm), weight (kg), education (no/primary school; secondary school; university/collage), physical activity (no/ < 2 h a week; 2–3 h a week; > 3 h a week), appearance and body functioning (very satisfied; medium satisfied; little satisfied; not satisfied at all), breast cancer treatment [surgery (breast conserving/ mastectomy), chemotherapy, radiation therapy, and hormonal therapy], relapse (yes/no/do not know), and symptoms (general pain; fatigue; lymphedema). The self-reported data on height and weight were used to calculate body mass index (BMI, kg/m^2^). Women without breast cancer received the questionnaire without questions on breast cancer diagnosis.

Information about age, detection mode, date of diagnosis, tumor diameter (mm), lymph node status (positive or negative), stage (I–IV, based on TNM classification) [19], and treatment was extracted from the Cancer Registry database. Self-reported information on treatment was  used if it was not available from the Cancer Registry database. In case of differences between the sources, the information from the database was used.

EQ-5D-5L questionnaire consisted of five dimensions (mobility, self-care, usual activities, pain/discomfort, and anxiety/depression) with five levels of severity (1–5), and a visual analogue scale (VAS) to report present health status (0–100) [20]. The response on VAS was included as the outcome in our analyses as a value of self-reported quality of life. The response to the questions regarding the five dimensions was used to obtain health utility values, representing the expected quality of life values on the day women responded to the questionnaire, which was approximated to one year period for the purposes of this study [20, 21]. We used ordinal regression to impute the missing numbers on the levels of severity, and available and imputed health utility values were compared (Online Appendix, Table A1). Women’s EQ-5D-5L responses were transformed to EQ-5D-3L responses using a cross-walk algorithm [21, 22], and health utility values were produced using the Danish value set for the EQ-5D-3L. We opted to use the Danish weights for the EQ-5D-3L to obtain the health utility values due to the absence of any Norwegian weights for the EQ-5D and because health and quality of life perceptions are comparable for Norway and Denmark [23].

### Statistical methods

Descriptive information was presented by detection mode and included means with standard deviations (SD) for continuous variables, median scores with interquartile ranges (IQR) for quality of life, and numbers with proportions for categorical variables. We used t-tests or analysis of variance (ANOVA) with the Bonferroni adjustment for multiple comparisons (level of significance post hoc < 0.001), nonparametric equality of medians test, and a chi-square test for comparisons. Sensitivity analyses were performed to compare the characteristics of the women who were and were not included in the analyses. To assess how quality of life was related to detection mode, two sets of regression analysis were performed. First, the association of the self-reported quality of life score (VAS, 0–100) and detection mode (women with symptomatic, screen-detected and interval cancer and women without breast cancer) was analysed using a median regression model adjusted for age, BMI, education, physical activity, appearance and body functioning, general pain, fatigue and lymphedema. A separate model for women with breast cancer also included time since diagnosis, stage at diagnosis, relapse and types of treatment as adjusting variables. The fulfilled assumptions of normal regression, linearity and independence of observations, as well as the large sample size, were the main reasons to choose median regression. Missing values of BMI (*n* = 164) were imputed for regression analyses using a linear regression model. Missing values for each categorical variable were indicated as a dummy variable and were included in all regression models. Second, health utility values were used as an outcome variable in the linear regression models, adjusted for all available variables, separately for all four groups of women and for women with breast cancer. Unadjusted and adjusted health utility values with standard errors (SE) were used to graphically present trends from the third to the 14th year since breast cancer diagnosis for women with breast cancer and trends by age for all groups. The adjusted health utility values were obtained using a margin function for time since diagnosis or age after performing a fully adjusted regression model. As the data were obtained from each woman only once, we have assumed that every woman’s health status in each year following can be approximated by the sample mean for a succession of different groups of women by detection mode. When calculating QALYs, we did not adjust for overall or breast cancer specific survival, but assumed that all women survived for at least 14 years following diagnosis and for at least 67–83 years, depending on the age of diagnosis. The QALYs from the third to the 14th year since breast cancer diagnosis were calculated based on the health utility values from women per year since diagnosis stratified by detection mode. Numbers of women per year since diagnosis or age were also presented (Online Appendix, Table A2). Stata MP Version 16.1 (Stata, Texas, College Station) was used for analyses, while MS Excel was used to graphically present the health utility values over time. *P* values < 0.05 were considered statistically significant.

## Results

We sent the questionnaire to 11,500 women (Fig. 1a, b). The response was not received from 6368 women, while 343 explicitly refused to participate, and six women died before receiving the questionnaire. We excluded 276 women with no information on quality of life and 20 women due to self-reported detrimental health conditions unrelated to breast cancer. The final study sample consisted of data from 4487 (39%, 4487/11,500; of the sample at recruitment) women; 1021 for symptomatic cancer, 1206 for screen-detected cancer, 1005 for interval cancer and 1255 for women without breast cancer.

Women included in the analyses, regardless of detection mode, were on average one year younger at recruitment and at diagnosis compared to those not included (*p* < 0.05 for all) (Table 1). Sensitivity analyses showed mean tumor diameter to be smaller and the proportion of lymph node positive tumors lower for women with symptomatic cancer included in the analyses, compared to those not included (20.3, SD: 13.1 mm versus 22.2, 16.2 mm; 27.0%, 276/1021 versus 30.8%, 455/1479, *p* < 0.05 for both). Results of sensitivity analyses of the tumours and the women included and not included in the analyses are shown in the Online Appendix (Table A3).

**Table 1:** Baseline characteristics of women included and not included in the analyses

Variable	Women included in the analyses (*n* = 4487)				Women not included in the analyses (*n* = 7013)			
	Symptomatic cancer (*n* = 1021)	Screen-detected cancer (*n* = 1206)	Interval cancer (*n* = 1005)	Without breast cancer (*n* = 1255)	Symptomatic cancer (*n* = 1479)	Screen-detected cancer (*n* = 1294)	Interval cancer (*n* = 1495)	Without breast cancer (*n* = 2745)
Age at recruitment Mean (SD), years	65.2 (6.8)	67.4 (6.3)*	67.4 (6.0)^#^	65.5 (7.4)^§&^	66.3 (7.0)^~^	68.3 (6.5)*^~^	68.1 (6.3)^#~^	66.6(8.2)^§&~^
Age at diagnosis Mean (SD), years	57.3 (6.2)	59.9 (5.7)*	59.6 (5.4)^#^		58.5 (6.2)^~^	60.4 (5.7)*^~^	60.0 (5.4)^#~^	
Time since diagnosis Mean (SD), years	8.0 (3.4)	7.6 (3.4)*	7.8 (3.5)		7.8 (3.3)	8.0 (3.4)^~^	8.1 (3.4)^#¤~^	
Tumor diameter								
Mean (SD), mm	20.3 (13.0)	17.2 (14.3)*	21.0 (13.5)^¤^		22.2 (16.2)^~^	16.9 (13.2)*	21.1 (13.7)^¤^	
Missing, *n*	264	30	72		389	36	102	
Positive lymph nodes, *n* (%)	276 (27.0)	349 (28.9)	258 (35.6)^#¤^		455 (30.8)^~^	373 (28.8)	522 (34.9)^#^	
Missing, *n*	382	18	31		555	29	44	
Stage at diagnosis								
I, *n* (%)	393 (38.5)	697 (57.8)*	375 (37.3)^¤^		487 (32.9)^~^	743 (57.4)*	569 (38.9)^#¤^	
II, *n* (%)	397 (38.9)	283 (23.5)*	443 (44.1)^#¤^		562 (38.0)	287 (22.2)*	615 (41.1)^¤^	
III, *n* (%)	149 (14.6)	194 (16.1)	132 (13.1)		272 (18.4)^~^	208 (16.1)	211 (14.1)^#^	
IV, *n* (%)	28 (2.7)	8 (0.7)	8 (0.8)		60 (4.1)	17 (1.3)	20 (1.3)	
Missing, *n*	54	24	47		98	39	80	
Body mass index, kg/m^2^, mean (SD)	25.7 (4.4)	26.4 (4.3)*	25.4 (4.2)^¤^	26.0 (4.4)^&§^				
Missing, *n*	29	41	29	65				
Education								
No or primary school, *n* (%)	150 (14.7)	231 (19.2)*	185 (18.4)	223 (17.8)				
Secondary school, *n* (%)	376 (36.8)	469 (38.9)	350 (34.8)	488 (38.9)				
University/college, *n* (%)	487 (47.7)	493 (40.9)*	467 (46.5)	538 (42.9)				
Missing, *n*	8	13	3	6				
Physical activity								
No or < 2 h a week, *n* (%)	189 (18.5)	218 (18.1)	131 (13.0)	166 (13.2)				
2–3 h a week, *n* (%)	389 (38.1)	524 (43.5)	429 (42.7)	509 (40.6)				
> 3 h a week, *n* (%)	433 (42.4)	449 (37.2)	440 (43.8)	569 (45.3)				
Missing, *n*	10	15	5	11				
Appearance and body functioning								
Very satisfied, *n* (%)	151 (14.8)	203 (16.8)	167 (16.6)	235 (18.7)				
Medium satisfied, *n* (%)	460 (45.1)	586 (48.6)	505 (50.3)	671 (53.5)				
Little satisfied, *n* (%)	224 (21.9)	257 (21.3)	205 (20.4)	197 (15.7)				
Not satisfied at all, *n* (%)	170 (16.7)	135 (11.2)	116 (11.5)	71 (5.7)^^&§^				
Missing, *n*	16	25	12	81				
Surgery	1004 (98.3)	1201 (99.6)	994 (98.9)					
Breast conserving, *n* (%)	524 (51.3)	861 (71.4)*	561 (55.8)^#¤^					
Mastectomy, *n* (%)	480 (47.0)	340 (28.2)*	433 (43.1)^¤^					
Missing, *n*	-	3	1					
Chemotherapy, *n* (%)	550 (53.9)	495 (41.0)*	573 (57.0)^¤^					
Radiation therapy, *n* (%)	815 (79.8)	1039 (86.2)*	829 (82.5)					
Hormonal therapy, *n* (%)	491 (48.1)	523 (43.4)*	513 (51.0)^¤^					
Relapse, *n* (%)	105 (10.3)	77 (6.4)	38 (3.8)	-				
General pain, *n* (%)	330 (32.3)	295 (24.5)*	282 (28.1)	300 (23.9)^^^				
Fatigue, *n* (%)	435 (42.6)	287 (32.1)*	345 (34.3)	111 (8.8)^^&§^				
Lymphedema, *n* (%)	163 (16.0)	154 (12.8)	141 (14.0)	14 (1.1)^^&§^				
Mobility, Mean (SD)	1.5 (0.8)	1.5 (0.8)	1.4 (0.8)^¤^	1.3 (0.7)				
Self-care, Mean (SD)	1.1 (0.4)	1.1 (0.3)	1.1 (0.3)	1.1 (0.3)				
Usual activities, Mean (SD)	1.7 (1.0)	1.5 (0.8)	1.6 (0.8)^#^	1.3 (0.6)^^&§^				
Pain/discomfort, Mean (SD)	2.2 (1.0)	2.0 (0.8)	2.1 (0.8)	2.0 (0.8)^^^				
Anxiety/depression Mean (SD)	1.7 (0.8)	1.5 (0.8)	1.5 (0.7)	1.4 (0.6)^^^				
Health utility value (0–1) Mean (SD)	0.77 (0.16)	0.81 (0.14)*	0.80 (0.13)^#^	0.83 (0.13)^^&§^				
Quality of life (0–100)								
Median (IQR)	60 (49–80)	70 (50–80)	70 (50–80)	80 (70–90)				

*SD* standard deviation, *IQR* interquartile range

*p < 0.05 for women with symptomatic cancer versus screen-detected cancer

^#^*p* < 0.05 for women with symptomatic cancer versus interval cancer

¤*p* < 0.05 for women with screen-detected cancer versus interval cancer

^*p* < 0.05 for women with symptomatic cancer versus women without breast cancer

^&^*p* < 0.05 for women with screen-detected cancer versus women without breast cancer

^§^*p* < 0.05 for women with interval cancer versus women without breast cancer

~*p* < 0.05 for corresponding included versus not included in the analyses

A two-sample t-test was used to compare means of continuous variables; a chi-square test was used to compare proportions of categorical variables

*P* < 0.001 between the groups for age recruitment and diagnosis, time since diagnosis, tumor diameter, body mass index and index values for one-way analysis of variance with Bonferroni adjustment for multiple comparisons

*P* < 0.05 between the groups for quality of life for nonparametric equality of medians test

Among the included women, the proportion of women with university/college education was higher for women with symptomatic compared to screen-detected cancer (47.7%, 487/1021 versus 40.9%, 493/1206; *p* < 0.05) (Table 1). The proportion of women who underwent breast conserving surgery was higher for women with screen-detected compared to symptomatic and interval cancer (71.4%, 861/1206 versus 51.3%, 524/1021 and 55.8%, 561/1005; *p* < 0.05), while the proportion of women who underwent chemotherapy, as well as hormonal therapy, was lower for those with screen-detected compared to symptomatic and interval cancer (41.0%, 495/1206; 43.4%, 523/1021 versus 53.9%, 550/1206; 48.1%, 491/1021 and 57.0%, 573/1005; 51.0%, 513/1005, respectively) (*p* < 0.05 for all).

Mean health utility value was 0.77 (SD 0.16) for women with symptomatic cancer, 0.81 (SD: 0.14) for women with screen-detected cancer, and 0.80 (SD: 0.13) for those with interval cancer (*p* < 0.05) (Table 1). Mean health utility value was higher for women without breast cancer (0.83, SD: 0.13) compared to women with screen-detected, symptomatic and interval cancer (*p* < 0.05 for all). Median values for quality of life were 60 (IQR: 49–80) for women with symptomatic breast cancer, 70 (IQR: 50-80) for those with screen-detected and interval cancer, and 80 (IQR: 70-90) for women without breast cancer.

In the adjusted regression analyses, women with screen-detected and interval cancer, and women without breast cancer had a 3.7 (95%CI 2.2–5.2), 2.3 (95%CI 0.7–3.8), and 4.8 (95%CI 3.3–6.4) higher median quality of life score, respectively, using symptomatic cancer as reference (Table 2). Factors associated with a higher median quality of life score included secondary school or university/college versus no/primary school and ≥ 2 hours a week versus < 2 hours a week of physical activity. Factors associated with a lower median quality of life score included non-satisfaction with appearance and body functioning, aging, general pain, fatigue and lymphedema.

**Table 2:** The associations of the median self-reported quality of life score (EQ-5D-5L, visual analogue scale, 0–100) and different factors among women with symptomatic breast cancer, women with screen-detected breast cancer, women with interval breast cancer, and women without breast cancer, 2006–2017

	Unadjusted (*n* = 4487)			Adjusted* (*n* = 4487)		
	Coefficient	95% Confidence Interval	*p* value	Coefficient	95% Confidence Interval	*p* value
Mode of detection						
Symptomatic cancer	Reference			Reference		
Screen-detected cancer	10.0	8.3 to 11.7	< 0.01	3.7	2.2 to 5.2	< 0.01
Interval cancer	10.0	8.2 to 11.8	< 0.01	2.3	0.7 to 3.8	< 0.01
Women without breast cancer	20.0	18.3 to 21.7	< 0.01	4.8	3.3 to 6.4	< 0.01
Age (years)	0.00	− 0.05 to 0.05	1.00	− 0.22	− 0.3 to − 0.1	< 0.01
Body mass index (kg/m^2^)	− 1.0	− 1.2 to − 0.9	< 0.01	− 0.1	− 0.3 to 0.1	0.22
Education						
No or primary school	Reference			Reference		
Secondary school	0.0	− 1.4 to 1.5	1.0	2.2	0.7 to 3.7	< 0.01
University/college	1.0	− 0.4 to 2.4	0.17	4.6	3.1 to 6.1	< 0.01
Missing	0.0	− 6.3 to 6.3	1.0	− 3.3	− 11.2 to 4.5	0.41
Physical activity						
No or < 2 h a week	Reference			Reference		
2–3 h a week	10.0	7.9 to 12.1	< 0.01	4.4	2.9 to 6.0	< 0.01
> 3 h a week	15.0	12.9 to 17.1	< 0.01	6.1	4.5 to 7.8	< 0.01
Missing	10.0	2.3 to 17.7	0.01	− 0.6	− 7.3 to 6.3	0.88
Appearance and body functioning						
Very satisfied	Reference			Reference		
Medium satisfied	− 15.0	− 17.3 to − 12.7	< 0.01	− 9.4	− 10.8 to − 7.9	< 0.01
Little satisfied	− 25.0	− 27.7 to − 22.3	< 0.01	− 15.6	− 17.4 to − 13.8	< 0.01
Not satisfied at all	− 45.0	− 48.2 to − 41.9	< 0.01	− 25.9	− 28.2 to − 23.7	< 0.01
Missing	− 10.0	− 15.1 to − 4.9	< 0.01	− 8.3	− 11.7 to − 4.8	< 0.01
General pain						
No	Reference			Reference		
Yes	− 18.0	− 20.8 to − 15.2	< 0.01	− 8.4	− 9.6 to − 7.1	< 0.01
Fatigue						
No	Reference			Reference		
Yes	− 30.0	− 31.0 to − 29.0	< 0.01	− 15.6	− 16.9 to − 14.3	< 0.01
Lymphedema						
No	Reference			Reference		
Yes	− 11.0	− 13.2 to − 8.8	< 0.01	− 3.9	− 5.7 to − 2.2	< 0.01

*Adjusted for age, body mass index, education, physical activity, appearance and body functioning, general pain, fatigue and lymphedema

**Fig. 2:**
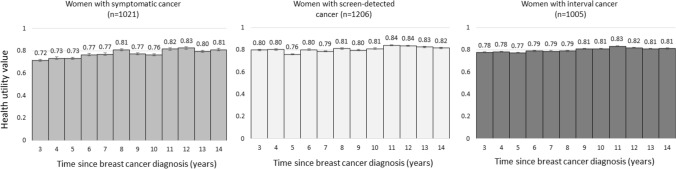
Health utility values from EQ-5D-5L for women with symptomatic, screen-detected and interval cancer over the time period from the third to the 14th year since diagnosis. Whiskers are standard errors

Health utility values of women with symptomatic, screen-detected and interval cancer tended to increase by time since diagnosis (Fig. 2). The lowest values were observed for the third year since diagnosis among women with symptomatic cancer (0.72, SE: 0.14) and the fifth year since diagnosis among women with screen-detected cancer (0.76, SE: 0.09) and interval cancer (0.77, SE: 0.05). The highest values were observed for the 11th year since diagnosis among women with screen-detected and interval cancer (0.84, SE: 0.09 and 0.83, SE: 0.07, respectively) and 12th year since diagnosis among women with symptomatic cancer (0.83, SE: 0.05).

Based on Table A4b and Fig. A1a, we estimated that a woman aged 50–69 years when her symptomatic cancer was diagnosed, and survived for at least 14 years since diagnosis would experience 9.5 QALYs as compared to a similarly aged woman with screen-detected and interval cancer surviving for the same time would experience 9.6 and 9.5 QALYs, respectively, during the period between the third and 14th year post-diagnosis.

The adjusted health utility values for all four groups did not show any specific trend by age, but generally tended to decrease from 0.81 (SE: 0.02)–0.82 (SE: 0.02) to 0.74 (SE: 0.02)–0.75 (SE: 0.02) between 58 and 82 years (Figure A1b).

## Discussion

Women with screen-detected cancer,  interval cancer and those without breast cancer had a 3.7, 2.3, and 4.8 higher median quality of life score, respectively, compared to women with symptomatic cancer. Based on the data from our study, women  with symptomatic, screen-detected and interval cancer would experience 9.5, 9.6 and 9.5 QALYs, respectively, between the third and the 14th year since diagnosis.

As far as we are aware, no studies have compared and published breast cancer survivors’ quality of life by detection mode. The studies comparing quality of life of breast cancer survivors of screening age and women without breast cancer showed inconsistent results [24–26]. QALYs for women with breast cancer associated with screening have never been compared based on data reported by the women, but rather from opinions of health care professionals or epidemiologists [27, 28]. An ideal study design would include a prospective study comparing quality of life and QALYs among women in the four groups over decades.

Breast cancer survivors who attended screening (screen-detected and interval cancer) had higher scores of quality of life compared to women with symptomatic cancer. This might imply that women attending screening are healthier and/or have a higher health consciousness and possibly more informed about benefits and harms of mammographic screening. However, women with symptomatic cancer consulted their general practitioner due to symptoms and might therefore also be better prepared for a cancer diagnosis as women with screen-detected cancer. Our finding of better quality of life among women with screen-detected compared to symptomatic cancer might indicate that women with screen-detected cancer were treated less aggressively compared to women with symptomatic cancer. Furthermore, women with symptomatic cancer who were included in our study had more favorable histopathologic tumor characteristics compared to those who were not included, which might have led to an overestimation of quality of life, and underestimation of the difference between women with symptomatic and screen-detected cancer. For women with screen-detected cancer, no differences in tumor characteristics were observed for  those who were included and not included in the study. Women with interval cancer showed higher quality of life scores compared to women with symptomatic cancer. This finding might suggest that women with interval cancer are more aware of the first symptoms of the disease due to information attained at screening. When compared to women without breast cancer, the quality of life scores for breast cancer survivors were lower, indicating the impact of the disease on the quality of life perception. It should be noted that our hypotheses, regarding higher quality of life of women without breast cancer compared to women with breast cancer and lower quality of life of women with symptomatic cancer compared to women with screen-detected and interval cancer, were confirmed.

The results on quality of life scores differed from the results on mean health utility values in the adjusted analyses, where the value was higher solely for women with screen-detected versus symptomatic cancer. Furthermore, trends over time showed consistently lower values for women with symptomatic cancer and women without breast cancer compared to screen-detected and interval cancer. This might indicate that women attending screening and diagnosed with breast cancer better manage the activities of daily living compared to women with symptomatic cancer and women without breast cancer. Possible reasons for this are not investigated in this study. We assume that the differences in VAS health status and QALYs across the groups are clinically meaningful as VAS indicates the participants’ perception of their general health condition while QALYs represent how the participants overcome daily living and are able to function. QALYs might thus be more clinically meaningful with regard to functioning compared to VAS. As far as we are aware, results of VAS and QALYs stratified by detection mode has not been presented elsewhere. The results of the study are thus important in the effort of balancing the benefits and harms of mammographic screening and the policy discussions about the cost-effectiveness of organized screening. We considered the results to be in favor of organized mammographic screening.

The proportion of women with university/college education was higher for women with symptomatic cancer compared to screen-detected cancer implying that women with higher education less frequently attend screening in Norway, which was also shown in a previous study using data from BreastScreen Norway [29]. Breast conserving surgery was more common, while chemotherapy and hormonal therapy were less common for women with screen-detected cancer compared to women with symptomatic and interval cancer in our study. This suggests that women with screen-detected cancer had an advantage in received treatment which might positively influenced their quality of life, compared to women with symptomatic and interval cancer. However, factors associated with changes in median quality of life included higher versus lower education and physical activity levels, non-satisfaction with appearance and body functioning, relapse, aging, general pain, fatigue and lymphedema. Breast cancer treatment was not associated with changes in quality of life or health utility values in our study. This might indicate that age, relapse and side-effects are more important determinants of quality of life in the long run than the treatment per se. Studies, not stratifying by detection mode, have shown that all these factors could affect quality of life among survivors [30–32].

### Study limitations

We did not use a breast cancer specific questionnaire, as the study did not intend to investigate disease specific domains of quality of life since we also included women without breast cancer. Possible confounding factors, such as race, ethnicity, histologic grade, receptor status, breast cancer subtypes and socio-economic status were not included as the data from the responding women and those available from the Cancer Registry of Norway was not complete and would have significantly reduced the sample size for the study if they had been included. These factors should be considered in future research. The response rates of women with interval cancer and women without breast cancer were lower than anticipated and were possibly affected by the restrictions associated with COVID-19 pandemic, which started March 13, 2020 in Norway. The response rate for women with screen-detected cancer was higher than for other groups. The reasons for this might be related to their confidence in mammographic screening and generally better compliance with the requests from the health care provider and the interest in assessing and reporting the results of the service and health condition in connection with the service.

The Cancer Registry of Norway receives information from the Population Registry regarding status, which includes date of death, immigration and emigration, every month. Some questionnaires were sent to women who had recently died and were not registered as dead in the Population Registry. Therefore, we used reported information from the next-of-kin of the women if they were dead within the time slot from extracting the study population to the time of receiving the questionnaire in the postbox. Further, an overestimation of quality of life might have occurred due to the age difference between the women who were included and not included in the study. However, as the women in all the groups were younger, the small differences did not influence the results. Women unable to read and write in Norwegian might have chosen not to participate, resulting in underestimation or overestimation of the effect, depending on numbers. A total of 217 women with screen-detected cancer were not included due to missing information about quality of life. The analyses of the baseline characteristics of women included versus not included due to missing information showed no significant differences (Table A5). Despite the relatively small differences in median quality of life between the groups, they might be clinically relevant, as they reflected the consistently higher mean health utility values for women with screen-detected or interval cancer compared to women with symptomatic cancer over time. Using a dummy variable for the missing values of the categorical variables might be associated with the biased impact estimates and potentially lead to misspecification of the functional form of the analysis model [33]. However, as the study was based on complete data of the existing factors, inclusion of all covariates in a correctly specified impact model could help increase the precision of the impact estimates. The cross-walk algorithm used to obtain health utility values might be related to restrictions on the range of scale possible for 5L values when mapping to a 3L value set, which would result in lower values for a five-level system compared to a three-level system [21, 22]. Another limitation of the cross-walk was associated with the data from a different country, which would result in both higher and lower values depending on health perceptions [22]. We did not include information on survival when calculating QALYs, as all women in our study population were alive at data collection, and the average life expectancy for Norwegian women, as of December 2017, is 84.3 years [34]. The latter was also the reason for non-adjusting for lead-time for women with screen-detected cancer [3].

Information bias was inevitable as the outcome was subjective and the assessment of the quality of life might have mostly been based on the presence or absence of breast cancer diagnosis. However, as the time since diagnosis was three to 14 years, the assessment of overall health and quality of life was also relevant for the participating women.

The ideal study design would have been a prospective study following up the women during a long term. We were not able to follow the women during 10 years to provide the information on their quality of life over time in this study, due to limited funding.

In conclusion, our study found that women with breast cancers detected by screening might have better quality of life compared to women with breast cancers detected due to symptoms.

## Supplementary Information

Below is the link to the electronic supplementary material.

Supplementary file1 (DOCX 392 KB)

## Data Availability

Data can be made available provided that the processing is in accordance with the principles set out in Article 5 of the General Data Protection Regulation (GDPR) and has legal basis in Articles 6 (1) (e) and 9 (2) (j) of the GDPR. In addition, the processing must have supplementary basis in Union or Member State law and ethical approval from the Regional Committees for Medical and Health Research Ethics. The data can only be made available to a third country or an international organisation, subject to the other provisions of GDPR, the conditions laid down in Chapter V is complied with.
