# Correction: Quality of life among women with symptomatic, screen‑detected, and interval breast cancer, and for women without breast cancer: a retrospective cross‑sectional study from Norway

**DOI:** 10.1007/s11136-025-03979-y

**Published:** 2025-06-27

**Authors:** Nataliia Moshina, Ragnhild S. Falk, Edoardo Botteri, Marthe Larsen, Lars A. Akslen, John A. Cairns, Solveig Hofvind

**Affiliations:** 1 Cancer Registry of Norway, Majorstuen, P.O. 5313, 0304 Oslo, Norway; 2 Oslo Centre for Biostatistics and Epidemiology, Oslo University Hospital, Oslo, Norway; 3 Cancer Registry of Norway, Oslo, Norway; 4 Centre for Cancer Biomarkers CCBIO, Department of Clinical Medicine, Section for Pathology, University of Bergen, Bergen, Norway; 5 Department of Health Services Research and Policy, London School of Hygiene and Tropical Medicine, London, UK; 6 Department of Health and Care Sciences, UiT The Arctic University of Norway, P.O. 6050, 9037 Tromso, Norway; 7 Department of Pathology, Haukeland University Hospital, Bergen, Norway

**Correction to: Quality of Life Research (2022) 31:1057–1068** 10.1007/s11136-021-03017-7

In the original publication of the article, partial information from women who refused to participate in the study was mistakenly included for comparison in Table 1, Table A3 and Results section.

Tables 1 and A3 and the text of the article related to the tables were now corrected after exclusion of this information. These changes did not affect the main results of the study.

The incorrect and corrected versions of Table 1 and Table A3 are provided in this correction. Also the sentence “Sensitivity analyses showed mean tumor diameter to be smaller and the proportion of lymph node positive tumors lower for women with symptomatic cancer included in the analyses, compared to those not included (20.3, SD: 13.1 mm versus 22.2, 16.2 mm; 27.0%, 276/1021 versus 30.8%, 455/1479, *p* < 0.05 for both).” in second paragraph of the Results section should have been “Sensitivity analyses showed mean tumor diameter to be smaller and the proportion of lymph node positive tumors lower for women with symptomatic cancer included in the analyses, compared to those not included (20.3, SD: 13*.0* mm versus *22.1,* SD: *15.5* mm; 27.0%, 276/1021 versus *31.2%, 439/1406*, p < 0.05 for both).”

Incorrect table

**Table 1:** Baseline characteristics of women included and not included in the analyses

Variable	Women included in the analyses (*n* = 4487)				Women not included in the analyses (*n* = 7013)			
	Symptomatic cancer (*n* = 1021)	Screen-detected cancer (*n* = 1206)	Interval cancer (*n* = 1005)	Without breast cancer (*n* = 1255)	Symptomatic cancer (*n* = 1479)	Screen-detected cancer (*n* = 1294)	Interval cancer (*n* = 1495)	Without breast cancer (*n* = 2745)
Age at recruitment Mean (SD), years	65.2 (6.8)	67.4 (6.3)*	67.4 (6.0)^#^	65.5 (7.4)^§&^	66.3 (7.0)^~^	68.3 (6.5)*^~^	68.1 (6.3)^#~^	66.6 (8.2)^§&~^
Age at diagnosis Mean (SD), years	57.3 (6.2)	59.9 (5.7)*	59.6 (5.4)^#^		58.5 (6.2)^~^	60.4 (5.7)*^~^	60.0 (5.4)^#~^	
Time since diagnosis Mean (SD), years	8.0 (3.4)	7.6 (3.4)*	7.8 (3.5)		7.8 (3.3)	8.0 (3.4)^~^	8.1 (3.4)^#¤~^	
Tumor diameter								
Mean (SD), mm	20.3 (13.0)	17.2 (14.3)*	21.0 (13.5)^¤^		22.2 (16.2)^~^	16.9 (13.2)*	21.1 (13.7)^¤^	
Missing, *n*	264	30	72		389	36	102	
Positive lymph nodes, *n* (%)	276 (27.0)	349 (28.9)	258 (35.6)^#¤^		455 (30.8)^~^	373 (28.8)	522 (34.9)^#^	
Missing, *n*	382	18	31		555	29	44	
Stage at diagnosis								
I, *n* (%)	393 (38.5)	697 (57.8)*	375 (37.3)^¤^		487 (32.9)^~^	743 (57.4)*	569 (38.9)^#¤^	
II, *n* (%)	397 (38.9)	283 (23.5)*	443 (44.1)^#¤^		562 (38.0)	287 (22.2)*	615 (41.1)^¤^	
III, *n* (%)	149 (14.6)	194 (16.1)	132 (13.1)		272 (18.4)^~^	208 (16.1)	211 (14.1)^#^	
IV, *n* (%)	28 (2.7)	8 (0.7)	8 (0.8)		60 (4.1)	17 (1.3)	20 (1.3)	
Missing, *n*	54	24	47		98	39	80	
Body mass index, kg/m^2^, mean (SD)	25.7 (4.4)	26.4 (4.3)*	25.4 (4.2)^¤^	26.0 (4.4)^&§^				
Missing, *n*	29	41	29	65				
Education								
No or primary school, *n* (%)	150 (14.7)	231 (19.2)*	185 (18.4)	223 (17.8)				
Secondary school, *n* (%)	376 (36.8)	469 (38.9)	350 (34.8)	488 (38.9)				
University/college, *n* (%)	487 (47.7)	493 (40.9)*	467 (46.5)	538 (42.9)				
Missing, *n*	8	13	3	6				
Physical activity								
No or < 2 h a week, *n* (%)	189 (18.5)	218 (18.1)	131 (13.0)	166 (13.2)				
2–3 h a week, *n* (%)	389 (38.1)	524 (43.5)	429 (42.7)	509 (40.6)				
> 3 h a week, *n* (%)	433 (42.4)	449 (37.2)	440 (43.8)	569 (45.3)				
Missing, *n*	10	15	5	11				
Appearance and body functioning								
Very satisfied, *n* (%)	151 (14.8)	203 (16.8)	167 (16.6)	235 (18.7)				
Medium satisfied, *n* (%)	460 (45.1)	586 (48.6)	505 (50.3)	671 (53.5)				
Little satisfied, *n* (%)	224 (21.9)	257 (21.3)	205 (20.4)	197 (15.7)				
Not satisfied at all, *n* (%)	170 (16.7)	135 (11.2)	116 (11.5)	71 (5.7)^^&§^				
Missing, *n*	16	25	12	81				
Surgery	1004 (98.3)	1201 (99.6)	994 (98.9)					
Breast conserving, *n* (%)	524 (51.3)	861 (71.4)*	561 (55.8)^#¤^					
Mastectomy, *n* (%)	480 (47.0)	340 (28.2)*	433 (43.1)^¤^					
Missing, *n*	–	3	1					
Chemotherapy, *n* (%)	550 (53.9)	495 (41.0)*	573 (57.0)^¤^					
Radiation therapy, *n* (%)	815 (79.8)	1039 (86.2)*	829 (82.5)					
Hormonal therapy, *n* (%)	491 (48.1)	523 (43.4)*	513 (51.0)^¤^					
Relapse, *n* (%)	105 (10.3)	77 (6.4)	38 (3.8)	–				
General pain, *n* (%)	330 (32.3)	295 (24.5)*	282 (28.1)	300 (23.9)^^^				
Fatigue, *n* (%)	435 (42.6)	287 (32.1)*	345 (34.3)	111 (8.8)^^&§^				
Lymphedema, *n* (%)	163 (16.0)	154 (12.8)	141 (14.0)	14 (1.1)^^&§^				
Mobility, Mean (SD)	1.5 (0.8)	1.5 (0.8)	1.4 (0.8)^¤^	1.3 (0.7)				
Self-care, Mean (SD)	1.1 (0.4)	1.1 (0.3)	1.1 (0.3)	1.1 (0.3)				
Usual activities, Mean (SD)	1.7 (1.0)	1.5 (0.8)	1.6 (0.8)^#^	1.3 (0.6)^^&§^				
Pain/discomfort, Mean (SD)	2.2 (1.0)	2.0 (0.8)	2.1 (0.8)	2.0 (0.8)^^^				
Anxiety/depression, Mean (SD)	1.7 (0.8)	1.5 (0.8)	1.5 (0.7)	1.4 (0.6)^^^				
Health utility value (0–1), Mean (SD)	0.77 (0.16)	0.81 (0.14)*	0.80 (0.13)^#^	0.83 (0.13)^^&§^				
Quality of life (0–100)								
Median (IQR)	60 (49–80)	70 (50–80)	70 (50–80)	80 (70–90)				

*SD* standard deviation, *IQR* interquartile range

*p < 0.05 for women with symptomatic cancer versus screen-detected cancer

^#^*p* < 0.05 for women with symptomatic cancer versus interval cancer

^¤^*p* < 0.05 for women with screen-detected cancer versus interval cancer

^^^*p* < 0.05 for women with symptomatic cancer versus women without breast cancer

^&^*p* < 0.05 for women with screen-detected cancer versus women without breast cancer

^§^*p* < 0.05 for women with interval cancer versus women without breast cancer

^~^*p* < 0.05 for corresponding included versus not included in the analyses

A two-sample t-test was used to compare means of continuous variables; a chi-square test was used to compare proportions of categorical variables

*p* < 0.001 between the groups for age recruitment and diagnosis, time since diagnosis, tumor diameter, body mass index and index values for one-way analysis of variance with Bonferroni adjustment for multiple comparisons

*p* < 0.05 between the groups for quality of life for nonparametric equality of medians test

Incorrect table

**Table A3:** Available baseline characteristics for the sample before recruitment, all excluded women and women included in the study

Variable	The sample before recruitment				All excluded women				Included women			
	Women with symptomatic cancer (n=2500)	Women with screen-detected cancer (n=2500)	Women with interval cancer (n=2500)	Women without breast cancer (n=4000)	Women with symptomatic cancer (n=1479)	Women with screen-detected cancer (n=1294)	Women with interval cancer (n=1495)	Women without breast cancer (n=2745)	Women with symptomatic cancer (n=1021)	Women with screen-detected cancer (n=1206)	Women with interval cancer (n=1005)	Women without breast cancer (n=1255)
Age at diagnosis, mean (SD), years	58.0 (6.2)	60.1 (5.7)^*^	59.9 (5.4)^#^		58.5 (6.2)	60.4 (5.7)^*^	60.0 (5.4)^#^		57.3 (6.2)^~^	59.9 (5.7)^*^^~^	59.6 (5.4)^#~^	
Age at recruitment, mean (SD), years	65.9 (6.9)	67.9 (6.5)^*^	67.8 (6.2)^#^	66.3 (8.0)^&^§^	66.3 (7.0)	68.3 (6.5)^*^	68.1 (6.3)^#^	66.6 (8.2)^&§^	65.2 (6.8)^~^	67.4 (6.3)^*^^~^	67.4 (6.0)^#~^	65.5 (7.4)^&§~^
Time since diagnosis, mean (SD), years	7.9 (3.4)	7.8 (3.4)	8.0 (3.4)^¤^		7.8 (3.3)	8.0 (3.4)	8.1 (3.4)^#^		8.0 (3.4)	7.6 (3.4)^*^^~^	7.8 (3.5)^~^	
Tumor diameter, mean (SD), mm	21.4 (15.0)	17.1 (13.7)^*^	21.1 (13.6)^¤^		22.2 (16.2)	16.9 (13.2)^*^	21.1 (13.7)^¤^		20.3 (13.0)^~^	17.2 (14.3)^*^	21.0 (13.5)^¤^	
Missing, n (%)	653 (26.1)	66 (2.6)	174 (7.0)		389 (26.3)	36 (2.8)	102 (6.8)		264 (25.9)	30 (2.5)	72 (7.2)	
Positive lymph nodes, n (%)	731 (29.2)	722 (28.9)^*^	880 (35.2)^#¤^		455 (30.8)	373 (28.8)	522 (24.9)^#^		276 (27.0)^~^	349 (28.9)	258 (35.6)^#¤~^	
Missing, n (%)	937 (37.5)	47 (1.9)	75 (3.0)		555 (37.5)	29 (2.2)	44 (2.9)		382 (37.4)	18 (1.5)	31 (3.1)	
Stage at diagnosis												
I, n (%)	880 (35.2)	1440 (57.6)^*^	944 (37.8)^¤^		487 (32.9)	743 (57.4)^*^	569 (38.9)^#¤^		393 (38.5)^~^	697 (57.8)^*^	375 (37.3)^¤^	
II, n (%)	959 (38.4)	570 (22.8)^*^	1058 (42.3)^¤^		562 (38.0)	287 (22.2)^*^	615 (41.1)^¤^		397 (38.9)	283 (23.5)^*^	443 (44.1)^#¤^	
III, n (%)	421 (16.8)	402 (16.1)	343 (13.7)		272 (18.4)	208 (15.1)	211 (14.1)^#^		149 (14.6)^~^	194 (16.1)	132 (13.1)	
IV, n (%)	88 (3.5)	25 (1.0)	28 (1.1)		60 (4.1)	17 (1.3)	20 (1.3)		28 (2.7)	8 (0.7)	8 (0.8)	
Missing, n (%)	152 (6.1)	63 (2.5)	127 (5.1)		98 (6.6)	39 (3.0)	80 (5.4)		54 (5.3)	24 (2.0)	47 (4.7)	
Surgery	2418 (96.7)	2484 (99.4)^*^	2470 (98.8)^#^		1414 (95.6)	1283 (99.2)^*^	1476 (98.7)^¤^		1004 (98.3)^~^	1201 (99.6)	994 (98.9)	
Breast conserving surgery, n (%)	1211 (48.4)	1750 (70.0)^*^	1367 (54.7)^#¤^		687 (46.5)	889 (68.7)^*^	806 (53.9)^#¤^		524 (51.3)^~^	861 (71.4)^*^	561 (55.8)^#¤^	
Mastectomy, n (%)	1207 (48.3)	734 (29.4)^*^	1103 (44.1)^¤^		727 (49.2)	394 (30.5)^*^	670 (44.8)^#¤^		480 (47.0)	340 (28.2)^*^	433 (43.1)^¤^	
Missing, n (%)	1 (0.0)	6 (0.2)	5 (0.2)		1 (0.1)	3 (0.2)	4 (0.3)		-	3 (2.5)	1 (0.0)	
Chemotherapy, n (%)	516 (20.6)	355 (14.2)^*^	550 (22.2)^¤^		292 (19.7)	170 (13.1)^*^	313 (20.9)^¤^		550 (53.9)^~^	495 (41.0)^*~^	573 (57.0)^¤ ~^	
Missing, n (%)	1286 (51.4)	1369 (54.8)	1281 (51.2)		789 (53.3)	714 (55.2)	751 (50.3)					
Radiation therapy, n (%)	1770 (70.8)	2006 (80.2)^*^	1908 (76.3)^#^		1027 (69.4)	1015 (78.4)^*^	1139 (76.2)^#^		815 (79.8)^~^	1039 (86.2)^*~^	829 (82.5)^~^	
Missing, n (%)	426 (17.0)	276 (11.0)	310 (12.4)		264 (17.9)	164 (12.7)	178 (11.9)					
Hormonal therapy, n (%)	543 (21.7)	490 (19.6)	593 (23.7)		313 (21.2)	256 (19.8)	362 (24.2)^#¤^		491 (48.1)^~^	523 (43.4)^*~^	513 (51.0)^¤~^	
Missing, n (%)	1241 (49.6)	1266 (50.6)	1225 (49.0)		747 (50.5)	654 (50.5)	705 (47.2)					

Abbreviations: SD – Standard deviation

*p<0.05 for women with symptomatic versus screen-detected cancer

^#^p<0.05 for women with symptomatic versus interval cancer

^¤^p<0.05 for women with screen-detected versus interval cancer

^^^p<0.05 for women with symptomatic cancer versus women without breast cancer

^&^p<0.05 for women with screen-detected cancer versus women without breast cancer

^§^p<0.05 for women with interval cancer versus women without breast cancer

^~^p<0.05 for corresponding included versus excluded

A two-sample t-test was used to compare means of continuous variables; a chi-square test was used to compare proportions of categorical variables

p<0.05 between the groups for age at diagnosis, age at recruitment, time since diagnosis and tumor diameter for one-way analysis of variance (ANOVA) with Bonferroni adjustment for multiple comparisons

Correct table

**Table 1:** Baseline characteristics of women included and not included in the analyses

Variable	Women included in the analyses (*n* = 4487)				Women not included in the analyses (*n* = 6670)^£^			
	Symptomatic cancer (*n* = 1021)	Screen-detected cancer (*n* = 1206)	Interval cancer (*n* = 1005)	Without breast cancer (*n* = 1255)	Symptomatic cancer (*n* = 1406)	Screen-detected cancer (*n* = 1211)	Interval cancer (*n* = 1427)	Without breast cancer (*n* = 2626)
Age at recruitment Mean (SD), years	65.2 (6.8)	67.4 (6.3)*	67.4 (6.0)^#^	65.5 (7.4)^§&^	66.1 (6.9)	68.3 (6.5)*	67.9 (6.3)^#^	66.6(8.2)^§&~^
Age at diagnosis Mean (SD), years	57.3 (6.2)	59.9 (5.7)*	59.6 (5.4)^#^		58.4 (6.2)	60.4 (5.7)*	59.9 (5.4)^#^	
Time since diagnosis Mean (SD), years	8.0 (3.4)	7.6 (3.4)*	7.8 (3.5)		7.7 (3.3)	8.0 (3.4)	8.1 (3.4)^#^	
Tumor diameter								
Mean (SD), mm	20.3 (13.0)	17.2 (14.3)*	21.0 (13.5)^¤^		22.1 (15.5)	16.9 (13.2)*	21.2 (13.8)^¤^	
Missing, *n*	264	30	72		356 (25.3)	36 (3.0)	99 (6.9)	
Positive lymph nodes, *n* (%)	276 (27.0)	349 (28.9)	258 (35.6)^#¤^		439 (31.2)	346 (28.6)	505 (35.4)^#^	
Missing, *n*	382	18	31		513 (36.5)	27 (2.2)	41 (2.9)	
Stage at diagnosis								
I, *n* (%)	393 (38.5)	697 (57.8)*	375 (37.3)^¤^		457 (32.5)	695 (57.4)*	537 (37.6)^#¤^	
II, *n* (%)	397 (38.9)	283 (23.5)*	443 (44.1)^#¤^		537 (38.5)	272 (22.5)*	588 (41.2)^¤^	
III, *n* (%)	149 (14.6)	194 (16.1)	132 (13.1)		261 (18.6)	190 (15.9)	206 (14.4)^#^	
IV, *n* (%)	28 (2.7)	8 (0.7)	8 (0.8)		58 (4.1)	16 (1.3)	19 (1.3)	
Missing, *n*	54	24	47		93 (6.6)	38 (3.1)	77 (5.4)	
Body mass index, kg/m^2^, mean (SD)	25.7 (4.4)	26.4 (4.3)*	25.4 (4.2)^¤^	26.0 (4.4)^&§^				
Missing, *n*	29	41	29	65				
Education								
No or primary school, *n* (%)	150 (14.7)	231 (19.2)*	185 (18.4)	223 (17.8)				
Secondary school, *n* (%)	376 (36.8)	469 (38.9)	350 (34.8)	488 (38.9)				
University/college, *n* (%)	487 (47.7)	493 (40.9)*	467 (46.5)	538 (42.9)				
Missing, *n*	8	13	3	6				
Physical activity								
No or < 2 h a week, *n* (%)	189 (18.5)	218 (18.1)	131 (13.0)	166 (13.2)				
2–3 h a week, *n* (%)	389 (38.1)	524 (43.5)	429 (42.7)	509 (40.6)				
> 3 h a week, *n* (%)	433 (42.4)	449 (37.2)	440 (43.8)	569 (45.3)				
Missing, *n*	10	15	5	11				
Appearance and body functioning								
Very satisfied, *n* (%)	151 (14.8)	203 (16.8)	167 (16.6)	235 (18.7)				
Medium satisfied, *n* (%)	460 (45.1)	586 (48.6)	505 (50.3)	671 (53.5)				
Little satisfied, *n* (%)	224 (21.9)	257 (21.3)	205 (20.4)	197 (15.7)				
Not satisfied at all, *n* (%)	170 (16.7)	135 (11.2)	116 (11.5)	71 (5.7)^^&§^				
Missing, *n*	16	25	12	81				
Surgery	1004 (98.3)	1201 (99.6)	994 (98.9)					
Breast conserving, *n* (%)	524 (51.3)	861 (71.4)*	561 (55.8)^#¤^					
Mastectomy, *n* (%)	480 (47.0)	340 (28.2)*	433 (43.1)^¤^					
Missing, *n*	–	3	1					
Chemotherapy, *n* (%)	550 (53.9)	495 (41.0)*	573 (57.0)^¤^					
Radiation therapy, *n* (%)	815 (79.8)	1039 (86.2)*	829 (82.5)					
Hormonal therapy, *n* (%)	491 (48.1)	523 (43.4)*	513 (51.0)^¤^					
Relapse, *n* (%)	105 (10.3)	77 (6.4)	38 (3.8)	–				
General pain, *n* (%)	330 (32.3)	295 (24.5)*	282 (28.1)	300 (23.9)^^^				
Fatigue, *n* (%)	435 (42.6)	287 (32.1)*	345 (34.3)	111 (8.8)^^&§^				
Lymphedema, *n* (%)	163 (16.0)	154 (12.8)	141 (14.0)	14 (1.1)^^&§^				
Mobility, Mean (SD)	1.5 (0.8)	1.5 (0.8)	1.4 (0.8)^¤^	1.3 (0.7)				
Self-care, Mean (SD)	1.1 (0.4)	1.1 (0.3)	1.1 (0.3)	1.1 (0.3)				
Usual activities, Mean (SD)	1.7 (1.0)	1.5 (0.8)	1.6 (0.8)^#^	1.3 (0.6)^^&§^				
Pain/discomfort, Mean (SD)	2.2 (1.0)	2.0 (0.8)	2.1 (0.8)	2.0 (0.8)^^^				
Anxiety/depression, Mean (SD)	1.7 (0.8)	1.5 (0.8)	1.5 (0.7)	1.4 (0.6)^^^				
Health utility value (0–1), Mean (SD)	0.77 (0.16)	0.81 (0.14)*	0.80 (0.13)^#^	0.83 (0.13)^^&§^				
Quality of life (0–100)								
Median (IQR)	60 (49–80)	70 (50–80)	70 (50–80)	80 (70–90)				

A two-sample t-test was used to compare means of continuous variables; a chi-square test was used to compare proportions of categorical variables

*p* < 0.001 between the groups for age recruitment and diagnosis, time since diagnosis, tumor diameter, body mass index and index values for one-way analysis of variance with Bonferroni adjustment for multiple comparisons

*p* < 0.05 between the groups for quality of life for nonparametric equality of medians test

*SD* standard deviation, *IQR* interquartile range

^£^women who explicitly refused to participate were excluded; *n* = 73 for women with symptomatic cancer, *n* = 83 for women with screen-detected cancer, *n* = 68 for women with interval cancer and *n* = 119 for women without breast cancer

*p < 0.05 for women with symptomatic cancer versus screen-detected cancer

^#^*p* < 0.05 for women with symptomatic cancer versus interval cancer

^¤^*p* < 0.05 for women with screen-detected cancer versus interval cancer

^^^*p* < 0.05 for women with symptomatic cancer versus women without breast cancer

^&^*p* < 0.05 for women with screen-detected cancer versus women without breast cancer

^§^*p* < 0.05 for women with interval cancer versus women without breast cancer

^~^*p* < 0.05 for corresponding included versus not included in the analyses

Correct table

**Table A3:** Available baseline characteristics for the sample before recruitment, excluded women and women included in the study

Variable	The sample before recruitment ^£^				Excluded women ^£^				Included women			
	Women with symptomatic cancer (n=2427)	Women with screen-detected cancer (n=2417)	Women with interval cancer (n=2432)	Women without breast cancer (n=3881)	Women with symptomatic cancer (n=1406)	Women with screen-detected cancer (n=1211)	Women with interval cancer (n=1427)	Women without breast cancer (n=2626)	Women with symptomatic cancer (n=1021)	Women with screen-detected cancer (n=1206)	Women with interval cancer (n=1005)	Women without breast cancer (n=1255)
Age at diagnosis, mean (SD), years	57.9 (6.2)	60.0 (5.7)^*^	59.8 (5.4)^#^		58.4 (6.2)	60.4 (5.7)^*^	59.9 (5.4)^#^		57.3 (6.2)^~^	59.9 (5.7)^*^^~^	59.6 (5.4)^#~^	
Age at recruitment, mean (SD), years	65.7 (6.9)	67.8 (6.5)^*^	67.7 (6.2)^#^	66.3 (8.0)^&^§^	66.1 (6.9)	68.3 (6.5)^*^	67.9 (6.3)^#^	66.6 (8.2)^&§^	65.2 (6.8)^~^	67.4 (6.3)^*^^~^	67.4 (6.0)^#~^	65.5 (7.4)^&§~^
Time since diagnosis, mean (SD), years	7.8 (3.4)	7.7 (3.4)	8.0 (3.4)^¤^		7.7 (3.3)	8.0 (3.4)	8.1 (3.4)^#^		8.0 (3.4)	7.6 (3.4)^*^^~^	7.8 (3.5)^~^	
Tumor diameter, mean (SD), mm	21.3 (14.5)	17.1 (13.8)^*^	21.1 (13.7)^¤^		22.1 (15.5)	16.9 (13.2)^*^	21.2 (13.8)^¤^		20.3 (13.0)^~^	17.2 (14.3)^*^	21.0 (13.5)^¤^	
Missing, n (%)	620 (25.6)	66 (2.7)	171 (7.0)		356 (25.3)	36 (3.0)	99 (6.9)		264 (25.9)	30 (2.5)	72 (7.2)	
Positive lymph nodes, n (%)	695 (28.8)	715 (29.5)^*^	863 (35.5)^#¤^		439 (31.2)	346 (28.6)	505 (35.4)^#^		276 (27.0)^~^	349 (28.9)	258 (35.6)^#¤~^	
Missing, n (%)	895 (36.9)	45 (1.9)	72 (3.0)		513 (36.5)	27 (2.2)	41 (2.9)		382 (37.4)	18 (1.5)	31 (3.1)	
Stage at diagnosis												
I, n (%)	850 (37.3)	1392 (59.1)^*^	912 (39.5)^¤^		457 (32.5)	695 (57.4)^*^	537 (37.6)^#¤^		393 (38.5)^~^	697 (57.8)^*^	375 (37.3)^¤^	
II, n (%)	934 (41.0)	555 (23.6)^*^	1031 (44.7)^¤^		537 (38.5)	272 (22.5)^*^	588 (41.2)^¤^		397 (38.9)	283 (23.5)^*^	443 (44.1)^#¤^	
III, n (%)	410 (18.0)	384 (16.3)	338 (14.6)		261 (18.6)	190 (15.9)	206 (14.4)^#^		149 (14.6)^~^	194 (16.1)	132 (13.1)	
IV, n (%)	86 (3.8)	24 (1.0)	27 (1.2)		58 (4.1)	16 (1.3)	19 (1.3)		28 (2.7)	8 (0.7)	8 (0.8)	
Missing, n (%)	147 (6.1)	62 (2.6)	124 (5.1)		93 (6.6)	38 (3.1)	77 (5.4)		54 (5.3)	24 (2.0)	47 (4.7)	
Surgery	2348 (96.7)	2401 (99.4)^*^	2402 (98.8)^#^		1344 (95.6)	1200 (99.1)^*^	1408 (98.7)^¤^		1004 (98.3)^~^	1201 (99.6)	994 (98.9)	
Breast conserving surgery, n (%)	1178 (48.5)	1700 (70.3)^*^	1331 (54.7)^#¤^		654 (46.5)	839 (69.3)^*^	770 (54.0)^#¤^		524 (51.3)^~^	861 (71.4)^*^	561 (55.8)^#¤^	
Mastectomy, n (%)	1170 (48.2)	701 (29.0)^*^	1071 (44.0)^¤^		690 (49.0)	361 (29.8)^*^	638 (44.7)^#¤^		480 (47.0)	340 (28.2)^*^	433 (43.1)^¤^	
Missing, n (%)	1 (0.0)	6 (0.2)	5 (0.2)		1 (0.1)	3 (0.3)	4 (0.3)		-	3 (2.5)	1 (0.0)	
Chemotherapy, n (%)	505 (20.8)	341 (14.1)^*^	539 (22.2)^¤^		281 (20.0)	156 (12.9)^*^	302 (21.2)^¤^		550 (53.9)^~^	495 (41.0)^*~^	573 (57.0)^¤ ~^	
Missing, n (%)	1252 (51.5)	1327 (54.9)	1249 (51.4)		755 (53.6)	678 (56.0)	720 (50.5)					
Radiation therapy, n (%)	1722 (71.0)	1940 (80.3)^*^	1866 (76.7)^#^		979 (69.6)	949 (78.4)^*^	1097 (76.9)^#^		815 (79.8)^~^	1039 (86.2)^*~^	829 (82.5)^~^	
Missing, n (%)	418 (17.2)	267 (11.1)	301 (12.4)		256 (18.2)	155 (12.6)	169 (11.9)					
Hormonal therapy, n (%)	516 (21.3)	467 (19.3)	579 (23.8)		286 (20.3)	233 (19.3)	348 (24.4)^#¤^		491 (48.1)^~^	523 (43.4)^*~^	513 (51.0)^¤~^	
Missing, n (%)	1214 (50.1)	1232 (51.0)	1201 (49.4)		721 (51.3)	620 (51.2)	681 (47.7)					

Abbreviations: SD – Standard deviation

£ women who explicitly refused to participate were excluded; n = 73 for women with symptomatic cancer, n = 83 for women with screen-detected cancer, n = 68 for women with interval cancer and n = 119 for women without breast cancer

*p<0.05 for women with symptomatic versus screen-detected cancer

# p<0.05 for women with symptomatic versus interval cancer

¤ p<0.05 for women with screen-detected versus interval cancer

^ p<0.05 for women with symptomatic cancer versus women without breast cancer

& p<0.05 for women with screen-detected cancer versus women without breast cancer

§ p<0.05 for women with interval cancer versus women without breast cancer

~ p<0.05 for corresponding included versus excluded

A two-sample t-test was used to compare means of continuous variables; a chi-square test was used to compare proportions of categorical variables

p<0.05 between the groups for age at diagnosis, age at recruitment, time since diagnosis and tumor diameter for one-way analysis of variance with Bonferroni adjustment for multiple comparisons

The original article has been corrected.

## Supplementary Information

Below is the link to the electronic supplementary material.

Supplementary file1 (DOCX 390 kb)

