# Supplementary material for: Quality of life among women with symptomatic, screen-detected, and interval breast cancer, and for women without breast cancer: a retrospective cross-sectional study from Norway
Source: Qual Life Res. 2021 Oct 26;31(4):1057–68. doi: 10.1007/s11136-021-03017-7 (PMC8547129; doi:10.1007/s11136-021-03017-7)

|  | Before imputation | | | |  | After imputation | | | |
| --- | --- | --- | --- | --- | --- | --- | --- | --- | --- |
| Variable | Women with symptomatic cancer (n=1021) | Women with screen-detected cancer (n=1206) | Women with interval cancer  (n=1005) | Women without breast cancer  (n=1255) |  | Women with symptomatic cancer (n=1021) | Women  with screen-detected cancer (n=1206) | Women with interval cancer  (n=1005) | Women without breast cancer (n=1255) |
| Health utility value, mean (SD) | 0.77 (0.18) | 0.81 (0.14) | 0.80 (0.14) | 0.83 (0.18) |  | 0.77 (0.16) | 0.81 (0.14) | 0.80 (0.13) | 0.83 (0.13) |
| Missing, n (%) | 12 (1.2) | 17 (1.4) | 22 (2.2) | 522 (41.6) |  |  |  |  |  |
|  |  |  |  |  |  |  |  |  |  |
| Mobility, mean (SD) | 1.5 (0.8) | 1.5 (0.8) | 1.4 (0.8) | 1.3 (0.7) |  | 1.5 (0.8) | 1.5 (0.8) | 1.4 (0.8) | 1.3 (0.7) |
| Missing, n (%) | 8 (0.8) | 6 (0.5) | 7 (0.7) | 9 (0.7) |  |  |  |  |  |
|  |  |  |  |  |  |  |  |  |  |
| Self-care, mean (SD) | 1.1 (0.4) | 1.1 (0.3) | 1.1 (0.3) | 1.1 (0.3) |  | 1.1 (0.4) | 1.1 (0.3) | 1.1 (0.3) | 1.1 (0.3) |
| Missing, n (%) | 3 (0.2) | 5 (0.4) | 4 (0.4) | 4 (0.3) |  |  |  |  |  |
|  |  |  |  |  |  |  |  |  |  |
| Usual activities, mean (SD) | 1.7 (1.0) | 1.5 (0.8) | 1.6 (0.8) | 1.3 (0.6) |  | 1.7 (1.0) | 1.5 (0.8) | 1.6 (0.8) | 1.3 (0.6) |
| Missing, n (%) | 5 (0.5) | 5 (0.4) | 7 (0.7) | 6 (0.5) |  |  |  |  |  |
|  |  |  |  |  |  |  |  |  |  |
| Pain/discomfort, mean (SD) | 2.2 (1.0) | 2.0 (0.8) | 2.1 (0.8) | 2.0 (0.8) |  | 2.2 (1.0) | 2.0 (0.8) | 2.1 (0.8) | 2.0 (0.8) |
| Missing, n (%) | 4 (0.4) | 9 (0.7) | 6 (0.6) | 5 (0.4) |  |  |  |  |  |
|  |  |  |  |  |  |  |  |  |  |
| Anxiety/depression, mean (SD) | 1.6 (0.8) | 1.5 (0.8) | 1.5 (0.7) | 1.4 (0.6) |  | 1.7 (0.8) | 1.5 (0.8) | 1.5 (0.7) | 1.4 (0.6) |
| Missing, n (%) | 12 (1.2) | 17 (1.4) | 21 (2.1) | 520 (41.4) |  |  |  |  |  |

**Appendix**Table A1. Comparison of mean health utility and domain values of EQ-5D-5L with standard deviation (SD) for women with symptomatic breast cancer, women with screen-detected breast cancer, women with interval breast cancer and women without breast cancer before and after imputation

Table A2. Numbers of women with symptomatic, screen-detected, interval breast cancer (and women without breast cancer included in the analyses stratified by time since breast cancer diagnosis and by age

| Time since diagnosis (years) | Women with symptomatic cancer | Women with screen-detected cancer | Women with interval cancer | Women without breast cancer | Total |
| --- | --- | --- | --- | --- | --- |
| 3 | 108 | 159 | 129 | - | 396 |
| 4 | 96 | 130 | 107 | - | 333 |
| 5 | 91 | 125 | 80 | - | 296 |
| 6 | 103 | 117 | 102 | - | 322 |
| 7 | 105 | 112 | 87 | - | 304 |
| 8 | 81 | 116 | 77 | - | 274 |
| 9 | 93 | 70 | 64 | - | 227 |
| 10 | 67 | 89 | 82 | - | 238 |
| 11 | 69 | 79 | 85 | - | 233 |
| 12 | 68 | 86 | 78 | - | 232 |
| 13 | 72 | 60 | 61 | - | 193 |
| 14 | 68 | 63 | 53 | - | 184 |
| Total | 1021 | 1206 | 1005 | - | 3232 |
| Age (years) |  |  |  |  |  |
| 53 | 20 | 3 | 0 | 7 | 30 |
| 54 | 19 | 9 | 6 | 37 | 71 |
| 55 | 47 | 17 | 12 | 61 | 137 |
| 56 | 25 | 32 | 23 | 55 | 135 |
| 57 | 45 | 29 | 9 | 56 | 139 |
| 58 | 31 | 29 | 30 | 54 | 144 |
| 59 | 52 | 37 | 25 | 53 | 167 |
| 60 | 38 | 48 | 40 | 64 | 190 |
| 61 | 42 | 39 | 31 | 54 | 166 |
| 62 | 58 | 48 | 50 | 69 | 225 |
| 63 | 74 | 44 | 44 | 47 | 209 |
| 64 | 52 | 56 | 68 | 53 | 229 |
| 65 | 67 | 60 | 49 | 56 | 232 |
| 66 | 41 | 67 | 63 | 44 | 215 |
| 67 | 33 | 78 | 66 | 47 | 224 |
| 68 | 39 | 60 | 54 | 52 | 205 |
| 69 | 40 | 69 | 50 | 49 | 208 |
| 70 | 43 | 67 | 54 | 57 | 221 |
| 71 | 40 | 66 | 48 | 45 | 199 |
| 72 | 38 | 60 | 57 | 46 | 200 |
| 73 | 41 | 67 | 58 | 45 | 211 |
| 74 | 33 | 50 | 48 | 36 | 167 |
| 75 | 25 | 40 | 40 | 33 | 138 |
| 76 | 21 | 35 | 23 | 23 | 102 |
| 77 | 18 | 38 | 14 | 30 | 100 |
| 78 | 11 | 22 | 17 | 26 | 76 |
| 79 | 12 | 19 | 10 | 12 | 53 |
| 80 | 9 | 7 | 6 | 19 | 41 |
| 81 | 5 | 7 | 4 | 13 | 29 |
| 82 | 1 | 3 | 4 | 8 | 16 |
| 83 | 2 | 0 | 2 | 4 | 8 |
| Total | 1206 | 1021 | 1005 | 1255 | 4487 |

Table A3. Available baseline characteristics for the sample before recruitment, all excluded women and women included in the study

|  | The sample before recruitment ^£^ | | | | Excluded women ^£^ | | | | Included women | | | |
| --- | --- | --- | --- | --- | --- | --- | --- | --- | --- | --- | --- | --- |
| Variable | Women  with symptomatic cancer  (n=2427) | Women with screen-detected cancer (n=2417) | Women with interval cancer  (n=2432) | Women without breast cancer  (n=3881) | Women  with symptomatic cancer  (n=1406) | Women with screen-detected cancer (n=1211) | Women with interval cancer  (n=1427) | Women without breast cancer  (n=2626) | Women  with symptomatic cancer  (n=1021) | Women with screen-detected cancer (n=1206) | Women with interval cancer  (n=1005) | Women without breast cancer  (n=1255) |
| Age at diagnosis, mean (SD), years | 57.9 (6.2) | 60.0 (5.7)^*^ | 59.8 (5.4)^#^ |  | 58.4 (6.2) | 60.4 (5.7)^*^ | 59.9 (5.4)^#^ |  | 57.3 (6.2)^~^ | 59.9 (5.7)^*~^ | 59.6 (5.4)^#~^ |  |
| Age at recruitment, mean (SD), years | 65.7 (6.9) | 67.8 (6.5)^*^ | 67.7 (6.2)^#^ | 66.3 (8.0)^&^§^ | 66.1 (6.9) | 68.3 (6.5)^*^ | 67.9 (6.3)^#^ | 66.6 (8.2)^&§^ | 65.2 (6.8)^~^ | 67.4 (6.3)^*~^ | 67.4 (6.0)^#~^ | 65.5 (7.4)^&§~^ |
| Time since diagnosis, mean (SD), years | 7.8 (3.4) | 7.7 (3.4) | 8.0 (3.4)^¤^ |  | 7.7 (3.3) | 8.0 (3.4) | 8.1 (3.4)^#^ |  | 8.0 (3.4) | 7.6 (3.4)^*~^ | 7.8 (3.5)^~^ |  |
|  |  |  |  |  |  |  |  |  |  |  |  |  |
| Tumor diameter, mean (SD), mm | 21.3 (14.5) | 17.1 (13.8)^*^ | 21.1 (13.7)^¤^ |  | 22.1 (15.5) | 16.9 (13.2)^*^ | 21.2 (13.8)^¤^ |  | 20.3 (13.0)^~^ | 17.2 (14.3)^*^ | 21.0 (13.5)^¤^ |  |
| Missing, n (%) | 620 (25.6) | 66 (2.7) | 171 (7.0) |  | 356 (25.3) | 36 (3.0) | 99 (6.9) |  | 264 (25.9) | 30 (2.5) | 72 (7.2) |  |
|  |  |  |  |  |  |  |  |  |  |  |  |  |
| Positive lymph nodes, n (%) | 695 (28.8) | 715 (29.5)^*^ | 863 (35.5)^#¤^ |  | 439 (31.2) | 346 (28.6) | 505 (35.4)^#^ |  | 276 (27.0)^~^ | 349 (28.9) | 258 (35.6)^#¤~^ |  |
| Missing, n (%) | 895 (36.9) | 45 (1.9) | 72 (3.0) |  | 513 (36.5) | 27 (2.2) | 41 (2.9) |  | 382 (37.4) | 18 (1.5) | 31 (3.1) |  |
|  |  |  |  |  |  |  |  |  |  |  |  |  |
| Stage at diagnosis |  |  |  |  |  |  |  |  |  |  |  |  |
| I, n (%) | 850 (37.3) | 1392 (59.1)^*^ | 912 (39.5)^¤^ |  | 457 (32.5) | 695 (57.4)^*^ | 537 (37.6)^#¤^ |  | 393 (38.5)^~^ | 697 (57.8)^*^ | 375 (37.3)^¤^ |  |
| II, n (%) | 934 (41.0) | 555 (23.6)^*^ | 1031 (44.7)^¤^ |  | 537 (38.5) | 272 (22.5)^*^ | 588 (41.2)^¤^ |  | 397 (38.9) | 283 (23.5)^*^ | 443 (44.1)^#¤^ |  |
| III, n (%) | 410 (18.0) | 384 (16.3) | 338 (14.6) |  | 261 (18.6) | 190 (15.9) | 206 (14.4)^#^ |  | 149 (14.6)^~^ | 194 (16.1) | 132 (13.1) |  |
| IV, n (%) | 86 (3.8) | 24 (1.0) | 27 (1.2) |  | 58 (4.1) | 16 (1.3) | 19 (1.3) |  | 28 (2.7) | 8 (0.7) | 8 (0.8) |  |
| Missing, n (%) | 147 (6.1) | 62 (2.6) | 124 (5.1) |  | 93 (6.6) | 38 (3.1) | 77 (5.4) |  | 54 (5.3) | 24 (2.0) | 47 (4.7) |  |
|  |  |  |  |  |  |  |  |  |  |  |  |  |
| Surgery | 2348 (96.7) | 2401 (99.4)^*^ | 2402 (98.8)^#^ |  | 1344 (95.6) | 1200 (99.1)^*^ | 1408 (98.7)^¤^ |  | 1004 (98.3)^~^ | 1201 (99.6) | 994 (98.9) |  |
| Breast conserving surgery, n (%) | 1178 (48.5) | 1700 (70.3)^*^ | 1331 (54.7)^#¤^ |  | 654 (46.5) | 839 (69.3)^*^ | 770 (54.0)^#¤^ |  | 524 (51.3)^~^ | 861 (71.4)^*^ | 561 (55.8)^#¤^ |  |
| Mastectomy, n (%) | 1170 (48.2) | 701 (29.0)^*^ | 1071 (44.0)^¤^ |  | 690 (49.0) | 361 (29.8)^*^ | 638 (44.7)^#¤^ |  | 480 (47.0) | 340 (28.2)^*^ | 433 (43.1)^¤^ |  |
| Missing, n (%) | 1 (0.0) | 6 (0.2) | 5 (0.2) |  | 1 (0.1) | 3 (0.3) | 4 (0.3) |  | - | 3 (2.5) | 1 (0.0) |  |
|  |  |  |  |  |  |  |  |  |  |  |  |  |
| Chemotherapy, n (%) | 505 (20.8) | 341 (14.1)^*^ | 539 (22.2)^¤^ |  | 281 (20.0) | 156 (12.9)^*^ | 302 (21.2)^¤^ |  | 550 (53.9)^~^ | 495 (41.0)^*~^ | 573 (57.0)^¤ ~^ |  |
| Missing, n (%) | 1252 (51.5) | 1327 (54.9) | 1249 (51.4) |  | 755 (53.6) | 678 (56.0) | 720 (50.5) |  |  |  |  |  |
|  |  |  |  |  |  |  |  |  |  |  |  |  |
| Radiation therapy, n (%) | 1722 (71.0) | 1940 (80.3)^*^ | 1866 (76.7)^#^ |  | 979 (69.6) | 949 (78.4)^*^ | 1097 (76.9)^#^ |  | 815 (79.8)^~^ | 1039 (86.2)^*~^ | 829 (82.5) ^~^ |  |
| Missing, n (%) | 418 (17.2) | 267 (11.1) | 301 (12.4) |  | 256 (18.2) | 155 (12.6) | 169 (11.9) |  |  |  |  |  |
|  |  |  |  |  |  |  |  |  |  |  |  |  |
| Hormonal therapy, n (%) | 516 (21.3) | 467 (19.3) | 579 (23.8) |  | 286 (20.3) | 233 (19.3) | 348 (24.4)^#¤^ |  | 491 (48.1)^~^ | 523 (43.4)^*~^ | 513 (51.0)^¤~^ |  |
| Missing, n (%) | 1214 (50.1) | 1232 (51.0) | 1201 (49.4) |  | 721 (51.3) | 620 (51.2) | 681 (47.7) |  |  |  |  |  |

Abbreviations: SD – Standard deviation

£ women who explicitly refused to participate were excluded; n=73 for women with symptomatic cancer, n=83 for women with screen-detected cancer, n=68 for women with interval cancer and n=119 for women without breast cancer

*p<0.05 for women with symptomatic versus screen-detected cancer

# p<0.05 for women with symptomatic versus interval cancer

¤ p<0.05 for women with screen-detected versus interval cancer

^ p<0.05 for women with symptomatic cancer versus women without breast cancer

& p<0.05 for women with screen-detected cancer versus women without breast cancer

§ p<0.05 for women with interval cancer versus women without breast cancer

~ p<0.05 for corresponding included versus excluded

A two-sample t-test was used to compare means of continuous variables; a chi-square test was used to compare proportions of categorical variables

p<0.05 between the groups for age at diagnosis, age at recruitment, time since diagnosis and tumor diameter for one-way analysis of variance with Bonferroni adjustment for multiple comparisons

Table A4:

a. The association of the self-reported quality of life score (visual analogue scale, 0-100) and related variables among women with symptomatic, screen-detected and interval breast cancer, 2006-2017

b. The association of the health utility values obtained from EQ-5D-5L and related variables among women with symptomatic, screen-detected and interval breast cancer, 2006-2017

c. The association of the health utility values obtained from EQ-5D-5L and related variables among women with symptomatic, screen-detected and interval breast cancer and women without breast cancer, 2006-2017

| a | Unadjusted (n=3232) | | | |  | Adjusted* (n=3232) | | | |
| --- | --- | --- | --- | --- | --- | --- | --- | --- | --- |
|  | Coefficient | 95% Confidence  Interval | | P-value |  | Coefficient | 95% Confidence  Interval | | P-value |
| Constant |  |  |  |  |  | 90.96 |  |  |  |
| Mode of detection |  |  |  |  |  |  |  |  |  |
| Symptomatic cancer | 60.0 |  |  |  |  | reference |  |  |  |
| Screen-detected cancer | 10.0 | 8.51 | 11.50 | <0.01 |  | 3.30 | 2.59 | 5.01 | <0.01 |
| Interval cancer | 10.0 | 8.45 | 11.56 | <0.01 |  | 2.14 | 0.40 | 3.88 | 0.02 |
|  |  |  |  |  |  |  |  |  |  |
| Age (years) | 0.13 | -0.01 | 0.26 | 0.06 |  | -0.20 | -0.32 | -0.07 | <0.01 |
|  |  |  |  |  |  |  |  |  |  |
| Body mass index (kg/m^2^) | -1.28 | -1.53 | -1.05 | <0.01 |  | -0.11 | -0.28 | 0.06 | 0.22 |
|  |  |  |  |  |  |  |  |  |  |
| Time since diagnosis (years) | 0.43 | -0.10 | 0.38 | 0.24 |  | 0.11 | -0.12 | 0.33 | 0.36 |
|  |  |  |  |  |  |  |  |  |  |
| Stage at diagnosis |  |  |  |  |  |  |  |  |  |
| I | 70.0 |  |  |  |  | reference |  |  |  |
| II | -5.0 | -7.23 | -2.77 | <0.01 |  | -1.30 | -2.97 | 0.36 | 0.13 |
| III | -8.0 | -11.00 | -5.03 | <0.01 |  | -0.64 | -3.03 | 1.76 | 0.60 |
| IV | -20.0 | -28.61 | -11.39 | <0.01 |  | 2.48 | -4.09 | 9.05 | 0.46 |
| Missing | 0 | -5.24 | 5.24 | 1.00 |  | 0.14 | -3.63 | 3.90 | 0.22 |
|  |  |  |  |  |  |  |  |  |  |
| Education | |  |  |  |  |  |  |  |  |
| No or primary school | 60.00 |  |  |  |  | reference |  |  |  |
| Secondary school | 5.0 | 2.39 | 7.61 | <0.01 |  | 2.23 | 0.24 | 4.23 | 0.03 |
| University/college | 10.0 | 7.46 | 12.54 | <0.01 |  | 5.24 | 3.28 | 7.21 | <0.01 |
| Missing | 0.0 | -10.67 | 10.67 | 1.0 |  | 0.72 | -10.25 | 11.78 | 0.94 |
|  |  |  |  |  |  |  |  |  |  |
| Physical activity |  |  |  |  |  |  |  |  |  |
| No or <2 hours a week | 50.0 |  |  |  |  | reference |  |  |  |
| 2-3 hours a week | 20.0 | 16.97 | 23.03 | <0.01 |  | 4.41 | 2.41 | 6.41 | <0.01 |
| >3 hours a week | 20.0 | 16.96 | 23.04 | <0.01 |  | 5.73 | 3.65 | 7.81 | <0.01 |
| Missing | 20.0 | 8.87 | 31.13 | <0.01 |  | -2.18 | -11.90 | 7.55 | 0.66 |
|  |  |  |  |  |  |  |  |  |  |
| Appearance and body functioning | |  |  |  |  |  |  |  |  |
| Very satisfied | 85.0 |  |  |  |  | reference |  |  |  |
| Medium satisfied | -15.0 | -16.72 | -13.28 | <0.01 |  | -10.36 | -12.33 | -8.39 | <0.01 |
| Little satisfied | -25.0 | -26.97 | -23.03 | <0.01 |  | -16.22 | -18.59 | -13.85 | <0.01 |
| Not satisfied at all | -45.0 | -47.22 | -42.78 | <0.01 |  | -26.69 | -29.49 | -23.89 | <0.01 |
| Missing | -15.0 | -19.89 | -10.11 | <0.01 |  | -10.85 | -17.10 | -4.60 | <0.01 |
|  |  |  |  |  |  |  |  |  |  |
| Relapse |  |  |  |  |  |  |  |  |  |
| No or do not know | 70.0 |  |  |  |  | reference |  |  |  |
| Yes | -10.0 | -12.78 | -7.22 | <0.01 |  | -5.92 | -8.63 | -3.21 | <0.01 |
|  |  |  |  |  |  |  |  |  |  |

| General pain |  |  |  |  |  |  |  |  |  |
| --- | --- | --- | --- | --- | --- | --- | --- | --- | --- |
| No | 70.0 |  |  |  |  | reference |  |  |  |
| Yes | -20.0 | -21.87 | -18.13 | <0.01 |  | -8.14 | -9.76 | -6.53 | <0.01 |
|  |  |  |  |  |  |  |  |  |  |
| Fatigue |  |  |  |  |  |  |  |  |  |
| No | 75.0 |  |  |  |  | reference |  |  |  |
| Yes | -25.0 | -26.75 | -23.26 | <0.01 |  | -15.19 | -16.75 | -16.63 | <0.01 |
|  |  |  |  |  |  |  |  |  |  |
| Lymphedema |  |  |  |  |  |  |  |  |  |
| No | 70.0 |  |  |  |  | reference |  |  |  |
| Yes | -15.0 | -17.15 | 12.85 | <0.01 |  | -3.47 | -5.55 | -1.40 | <0.01 |
|  |  |  |  |  |  |  |  |  |  |
| Surgery |  |  |  |  |  |  |  |  |  |
| Breast conserving surgery | 70.0 |  |  |  |  | reference |  |  |  |
| Mastectomy | -3.0 | -4.89 | -1.11 | <0.01 |  | 0.30 | -1.59 | 2.19 | 0.76 |
| Missing | 0.0 | -26.17 | 26.17 | 1.00 |  | 10.23 | -9.53 | 29.99 | 0.31 |
|  |  |  |  |  |  |  |  |  |  |
| Chemotherapy |  |  |  |  |  |  |  |  |  |
| No | 70.0 |  |  |  |  | reference |  |  |  |
| Yes | -5.0 | -6.97 | -3.03 | <0.01 |  | -0.05 | -1.62 | 1.52 | 0.76 |
|  |  |  |  |  |  |  |  |  |  |
| Radiation therapy |  |  |  |  |  |  |  |  |  |
| No | 70.0 |  |  |  |  | reference |  |  |  |
| Yes | 0.0 | -3.49 | 3.49 | 1.00 |  | 2.04 | -0.23 | 4.32 | 0.08 |
|  |  |  |  |  |  |  |  |  |  |
| Hormonal therapy |  |  |  |  |  |  |  |  |  |
| No | 70.0 |  |  |  |  | reference |  |  |  |
| Yes | -3.0 | -4.95 | -1.07 | <0.01 |  | -0.45 | -1.92 | 1.01 | 0.54 |
|  |  |  |  |  |  |  |  |  |  |
| b | Unadjusted (n=3232) | | | |  | Adjusted (n=3232) | | | |
|  | Coefficient | 95% Confidence  Interval | | P-value |  | Coefficient | 95% Confidence  Interval | | P-value |
| Constant |  |  |  |  |  | 0.88 |  |  |  |
| Mode of detection |  |  |  |  |  |  |  |  |  |
| Symptomatic cancer | 0.77 |  |  |  |  | reference |  |  |  |
| Screen-detected cancer | 0.03 | 0.02 | 0.05 | <0.01 |  | 0.01 | 0.00 | 0.02 | 0.05 |
| Interval cancer | 0.03 | 0.01 | 0.04 | <0.01 |  | 0.01 | 0.00 | 0.02 | 0.19 |
|  |  |  |  |  |  |  |  |  |  |
| Age (years) | 0.003 | 0.002 | 0.004 | <0.01 |  | -0.00 | -0.00 | 0.00 | 0.28 |
|  |  |  |  |  |  |  |  |  |  |
| Body mass index (kg/m^2^) | -0.01 | -0.01 | 0.00 | <0.01 |  | 0.00 | -0.00 | 0.00 | 0.95 |
|  |  |  |  |  |  |  |  |  |  |
| Time since diagnosis (years) | 0.01 | 0.004 | 0.006 | <0.01 |  | 0.002 | 0.001 | 0.003 | 0.01 |
|  |  |  |  |  |  |  |  |  |  |
| Stage at diagnosis |  |  |  |  |  |  |  |  |  |
| I | 0.81 |  |  |  |  | reference |  |  |  |
| II | -0.03 | -0.05 | -0.02 | <0.01 |  | -0.01 | -0.02 | -0.00 | 0.02 |
| III | -0.04 | -0.06 | -0.03 | <0.01 |  | -0.00 | -0.02 | 0.01 | 0.57 |
| IV | -0.06 | -0.11 | -0.02 | <0.01 |  | -0.02 | -0.06 | 0.02 | 0.30 |
| Missing | -0.03 | -0.06 | -0.01 | 0.02 |  | -0.02 | -0.04 | 0.00 | 0.08 |
|  |  |  |  |  |  |  |  |  |  |
| Education | |  |  |  |  |  |  |  |  |
| No or primary school | 0.78 |  |  |  |  | reference |  |  |  |
| Secondary school | -0.00 | -0.01 | 0.01 | 0.94 |  | 0.01 | -0.00 | 0.02 | 0.22 |
| University/college | 0.03 | 0.02 | 0.04 | <0.01 |  | 0.02 | 0.01 | 0.03 | <0.01 |
| Missing | -0.02 | -0.08 | 0.04 | 0.48 |  | -0.05 | -0.12 | 0.01 | 0.10 |
|  |  |  |  |  |  |  |  |  |  |

| Physical activity |  |  |  | |  |  |  |  |  |  |
| --- | --- | --- | --- | --- | --- | --- | --- | --- | --- | --- |
| No or <2 hours a week | 0.74 |  |  | |  |  | reference |  |  |  |
| 2-3 hours a week | 0.05 | 0.04 | 0.07 | | <0.01 |  | 0.02 | 0.01 | 0.03 | <0.01 |
| >3 hours a week | 0.08 | 0.06 | 0.09 | | <0.01 |  | 0.02 | 0.01 | 0.04 | <0.01 |
| Missing | 0.04 | -0.01 | 0.09 | | 0.12 |  | -0.00 | -0.06 | 0.01 | 0.89 |
|  |  |  |  | |  |  |  |  |  |  |
| Appearance and body functioning | |  |  | |  |  |  |  |  |  |
| Very satisfied | 0.89 |  |  | |  |  | reference |  |  |  |
| Medium satisfied | -0.08 | -0.09 | -0.07 | | <0.01 |  | -0.05 | -0.06 | -0.04 | <0.01 |
| Little satisfied | -0.14 | -0.16 | -0.13 | | <0.01 |  | -0.09 | -0.10 | -0.07 | <0.01 |
| Not satisfied at all | -0.24 | -0.25 | -0.22 | | <0.01 |  | -0.15 | -0.17 | -0.14 | <0.01 |
| Missing | -0.09 | -0.13 | -0.05 | | <0.01 |  | -0.04 | -0.08 | -0.00 | 0.03 |
|  |  |  |  | |  |  |  |  |  |  |
| Relapse |  |  |  | |  |  |  |  |  |  |
| No or do not know | 0.80 |  |  | |  |  | reference |  |  |  |
| Yes | -0.05 | -0.07 | -0.03 | | <0.01 |  | -0.02 | -0.04 | -0.01 | <0.01 |
|  |  |  |  | |  |  |  |  |  |  |
| General pain |  |  |  | |  |  |  |  |  |  |
| No | 0.83 |  |  | |  |  | reference |  |  |  |
| Yes | -0.13 | -0.14 | -0.12 | | <0.01 |  | -0.07 | -0.08 | -0.06 | <0.01 |
|  |  |  |  | |  |  |  |  |  |  |
| Fatigue |  |  |  | |  |  |  |  |  |  |
| No | 0.84 |  |  | |  |  | reference |  |  |  |
| Yes | -0.14 | -0.15 | -0.13 | | <0.01 |  | -0.08 | -0.09 | -0.07 | <0.01 |
|  |  |  |  | |  |  |  |  |  |  |
| Lymphedema |  |  |  | |  |  |  |  |  |  |
| No | 0.80 |  |  | |  |  | reference |  |  |  |
| Yes | -0.07 | -0.08 | -0.06 | | <0.01 |  | -0.02 | -0.03 | -0.01 | <0.01 |
|  |  |  |  | |  |  |  |  |  |  |
| Surgery |  |  |  | |  |  |  |  |  |  |
| Breast conserving surgery | 0.80 |  |  | |  |  | reference |  |  |  |
| Mastectomy | -0.02 | -0.03 | -0.01 | | <0.01 |  | 0.01 | -0.01 | 0.02 | 0.37 |
| Missing | 0.08 | -0.06 | 0.23 | | 0.24 |  | 0.12 | 0.01 | 0.24 | 0.03 |
|  |  |  |  | |  |  |  |  |  |  |
| Chemotherapy |  |  |  | |  |  |  |  |  |  |
| No | 0.81 |  |  | |  |  | reference |  |  |  |
| Yes | -0.04 | -0.05 | -0.03 | | <0.01 |  | -0.01 | -0.02 | 0.00 | 0.18 |
|  |  |  |  | |  |  |  |  |  |  |
| Radiation therapy |  |  |  | |  |  |  |  |  |  |
| No | 0.79 |  |  | |  |  | reference |  |  |  |
| Yes | -0.00 | -0.02 | 0.01 | | 0.67 |  | 0.01 | -0.02 | 0.00 | 0.09 |
|  |  |  |  | |  |  |  |  |  |  |
| Hormonal therapy |  |  |  | |  |  |  |  |  |  |
| No | 0.80 |  |  | |  |  | reference |  |  |  |
| Yes | -0.01 | -0.02 | -0.00 | | 0.02 |  | 0.00 | -0.01 | 0.01 | 0.66 |
|  |  |  |  | |  |  |  |  |  |  |
|  |  |  |  | |  |  |  |  |  |  |
| c | Unadjusted (n=4487) | | | | |  | Adjusted (n=4487) | | | |
|  | Coefficient | 95% Confidence  Interval | | P-value | |  | Coefficient | 95% Confidence  Interval | | P-value |
| Constant |  |  |  |  | |  | 0.89 |  |  |  |
| Mode of detection |  |  |  |  | |  |  |  |  |  |
| Symptomatic cancer | 0.77 |  |  |  | |  | reference |  |  |  |
| Screen-detected cancer | 0.03 | 0.02 | 0.04 | <0.01 | |  | 0.01 | 0.00 | 0.02 | 0.01 |
| Interval cancer | 0.03 | 0.01 | 0.04 | <0.01 | |  | 0.01 | -0.00 | 0.02 | 0.13 |
| Women without breast cancer | 0.06 | 0.05 | 0.07 | <0.01 | |  | 0.00 | -0.01 | 0.01 | 0.76 |
|  |  |  |  |  | |  |  |  |  |  |
| Age (years) | 0.002 | 0.001 | 0.002 | <0.01 | |  | -0.00 | -0.00 | 0.00 | 0.19 |
|  |  |  |  |  | |  |  |  |  |  |
| Body mass index (kg/m^2^) | -0.01 | -0.01 | -0.00 | <0.01 | |  | 0.00 | -0.00 | 0.00 | 0.35 |
|  |  |  |  |  | |  |  |  |  |  |

| Education | |  |  |  |  |  |  |  |  |
| --- | --- | --- | --- | --- | --- | --- | --- | --- | --- |
| No or primary school | 0.79 |  |  |  |  | reference |  |  |  |
| Secondary school | 0.01 | -0.00 | 0.02 | 0.17 |  | 0.01 | 0.00 | 0.02 | 0.03 |
| University/college >3 years | 0.03 | 0.02 | 0.05 | <0.01 |  | 0.03 | 0.02 | 0.04 | <0.01 |
| Missing | -0.01 | -0.06 | 0.04 | 0.73 |  | -0.04 | -0.09 | 0.01 | 0.12 |
|  |  |  |  |  |  |  |  |  |  |
| Physical activity |  |  |  |  |  |  |  |  |  |
| No or <2 hours a week | 0.75 |  |  |  |  | reference |  |  |  |
| 2-3 hours a week | 0.05 | 0.04 | 0.06 | <0.01 |  | 0.02 | 0.01 | 0.03 | <0.01 |
| >3 hours a week | 0.08 | 0.07 | 0.09 | <0.01 |  | 0.03 | 0.02 | 0.04 | <0.01 |
| Missing | 0.05 | 0.01 | 0.09 | 0.03 |  | -0.00 | -0.05 | 0.04 | 0.90 |
|  |  |  |  |  |  |  |  |  |  |
| Appearance and body functioning | |  |  |  |  |  |  |  |  |
| Very satisfied | 0.90 |  |  |  |  | reference |  |  |  |
| Medium satisfied | -0.08 | -0.09 | -0.06 | <0.01 |  | -0.06 | -0.06 | -0.05 | <0.01 |
| Little satisfied | -0.14 | -0.15 | -0.13 | <0.01 |  | -0.09 | -0.10 | -0.08 | <0.01 |
| Not satisfied at all | -0.24 | -0.25 | -0.22 | <0.01 |  | -0.16 | -0.17 | -0.14 | <0.01 |
| Missing | -0.06 | -0.08 | -0.04 | <0.01 |  | -0.03 | -0.06 | -0.01 | <0.01 |
|  |  |  |  |  |  |  |  |  |  |
| General pain |  |  |  |  |  |  |  |  |  |
| No | 0.84 |  |  |  |  | reference |  |  |  |
| Yes | -0.13 | -0.14 | -0.12 | <0.01 |  | -0.08 | -0.09 | -0.07 | <0.01 |
|  |  |  |  |  |  |  |  |  |  |
| Fatigue |  |  |  |  |  |  |  |  |  |
| No | 0.84 |  |  |  |  | reference |  |  |  |
| Yes | -0.14 | -0.15 | -0.13 | <0.01 |  | -0.08 | -0.09 | -0.08 | <0.01 |
|  |  |  |  |  |  |  |  |  |  |
| Lymphedema |  |  |  |  |  |  |  |  |  |
| No | 0.81 |  |  |  |  | reference |  |  |  |
| Yes | -0.08 | -0.09 | -0.06 | <0.01 |  | -0.02 | -0.03 | -0.01 | <0.01 |

^*^ a and b: Adjusted for age, body mass index, time since diagnosis, stage at diagnosis education, physical activity, appearance and body functioning, relapse, general pain, fatigue, lymphedema and types of treatment;

c: Adjusted for age, body mass index, education, physical activity, appearance and body functioning, general pain, fatigue and lymphedema

Interpretation:

1. In the adjusted regression analyses including solely women with breast cancer, women with screen-detected cancer and women with interval cancer had a higher median quality of life score (3.3; 95%CI 2.6-5.0; and 2.1; 95%CI 0.4-3.9, respectively) compared to women with symptomatic cancer. The factors associated with an increase in a median quality of life included education (secondary school or university/college compared to no/primary school) and physical activity (≥2 hours a week compared to <2 hours a week). The factors associated with a decrease in a median quality of life score included non-satisfaction with appearance and body functioning, relapse, aging, general pain, fatigue and lymphedema.
2. In the adjusted regression analyses including solely women with breast cancer, no differences were found in health utility values for women with screen-detected cancer and women with interval cancer compared to women with symptomatic cancer. The factors associated with an increase in health utility value included education (university/college compared to no/primary school), physical activity (≥2 hours a week compared to <2 hours a week), and time since breast cancer diagnosis. The factors associated with a decrease in health utility value included stage II breast cancer (compared to stage I breast cancer), non-satisfaction with appearance and body functioning, relapse, aging, general pain, fatigue and lymphedema.
3. In the adjusted regression analyses including all four groups of women, women with screen-detected cancer had a higher mean health utility value (0.01; 95% CI 0.00-0.02) compared to women with symptomatic cancer. The factors associated with an increase in mean health utility value included education (university/college >3 years compared to no/primary school) and physical activity (≥2 hours a week compared to <2 hours a week). The factors associated with a decrease in health utility value included non-satisfaction with appearance and body functioning, general pain, fatigue and lymphedema.

| Table A5. Characteristics of the women who were included in the analyses and women who were not included due to missing information about quality of life | | | | | | | | |
| --- | --- | --- | --- | --- | --- | --- | --- | --- |
|  | Women included in the analyses | | | | Women not included in the analyses due to missing information about quality of life | | | |
| Variable | With symptomatic cancer  (n=1021) | With screen-detected cancer (n=1206) | With interval cancer  (n=1005) | Without breast cancer  (n=1255) | With symptomatic cancer  (n=20) | With screen-detected cancer  (n=217) | With interval cancer  (n=17) | Without breast cancer  (n=22) |
| Age at diagnosis, mean (SD), years | 57.3 (6.2) | 59.9 (5.7) | 59.6 (5.4) |  | 56.0 (6.9) | 59.9 (5.8) | 57.8 (5.2) |  |
| Age at recruitment, mean (SD), years | 65.2 (6.8) | 67.4 (6.3) | 67.4 (6.0) | 65.5 (7.4) | 63.1 (7.6) | 67.7 (6.7) | 66.5 (5.6) | 69.5 (7.6) |
| Time since diagnosis, mean (SD), years | 8.0 (3.4) | 7.6 (3.4) | 7.8 (3.5) |  | 7.1 (3.3) | 7.7 (3.4) | 8.9 (2.9) |  |
| Body mass index, mean (SD), kg/m^2^ | 25.7 (4.4) | 26.4 (4.3) | 25.4 (4.2) | 26.0 (4.4) | 26.0 (5.9) | 27.1 (4.8) | 26.3 (5.2) | 27.7 (4.4) |
| Missing, n | 29 | 41 | 29 | 65 | 5 | 187 | 7 | 3 |
|  |  |  |  |  |  |  |  |  |
| Stage at diagnosis |  |  |  |  |  |  |  |  |
| I, n (%) | 393 (38.5) | 697 (57.8) | 375 (37.3) |  | 7 (35.0) | 130 (59.9) | 7 (41.2) |  |
| II, n (%) | 397 (38.9) | 283 (23.5) | 443 (44.1) |  | 9 (45.0) | 47 (21.7) | 8 (47.1) |  |
| III, n (%) | 149 (14.6) | 194 (16.1) | 132 (13.1) |  | 1 (5.0) | 36 (16.6) | 2 (11.8) |  |
| IV, n (%) | 28 (2.7) | 8 (0.7) | 8 (0.8) |  | 1 (5.0) | - | - |  |
| Missing, n | 54 | 24 | 47 |  | 2 | 4 | - |  |
|  |  |  |  |  |  |  |  |  |
| Tumor diameter, mean (SD), mm | 20.3 (13.0) | 17.2 (14.3) | 21.0 (13.5) |  | 20.7 (5.9) | 27.1 (4.8)^*^ | 22.8 (13.5) |  |
| Missing, n | 264 | 30 | 72 |  | 4 | 5 | 1 |  |
|  |  |  |  |  |  |  |  |  |
| Positive lymph nodes, n (%) | 276 (27.0) | 349 (28.9) | 258 (35.6) |  | 3 (15.0) | 65 (30.0) | 6 (35.3) |  |
| Missing, n | 382 | 18 | 31 |  | 6 | 4 | - |  |
|  |  |  |  |  |  |  |  |  |
| Surgery | 1004 (98.3) | 1201 (99.6) | 994 (98.9) |  | 19 (95.0) | 215 (99.1) | 17 (100.0) |  |
| Breast conserving surgery, n (%) | 524 (51.3) | 861 (71.4) | 561 (55.8) |  | 11 (55.0) | 150 (69.1) | 7 (41.2) |  |
| Mastectomy, n (%) | 480 (47.0) | 340 (28.2) | 433 (43.1) |  | 8 (40.0) | 65 (30.0) | 10 (58.8) |  |
| Missing, n | - | 3 | 1 |  | - | - | - |  |
|  |  |  |  |  |  |  |  |  |
| Chemotherapy, n (%) | 550 (53.9) | 495 (41.0) | 573 (57.0) |  | 10 (50.0) | 39 (18.0) | 9 (52.9) |  |
| Radiation therapy, n (%) | 815 (79.8) | 1039 (86.2) | 829 (82.5) |  | 15 (75.0) | 178 (82.0) | 13 (76.5) |  |
| Hormonal therapy, n (%) | 491 (48.1) | 523 (43.4) | 513 (51.0) |  | 11 (55.0) | 51 (23.5) | 7 (41.2) |  |
|  |  |  |  |  |  |  |  |  |
| Education |  |  |  |  |  |  |  |  |
| No or primary school, n (%) | 150 (14.7) | 231 (19.2) | 185 (18.4) | 223 (17.8) | 3 (15.0) | 7 (3.2) | 2 (11.8) | 8 (36.4) |
| Secondary school, n (%) | 376 (36.8) | 469 (38.9) | 350 (34.8) | 488 (38.9) | 7 (35.0) | 9 (4.2) | 6 (35.3) | 6 (27.3) |
| University/college, n (%) | 487 (47.7) | 493 (40.9) | 467 (46.5) | 538 (42.9) | 6 (30.0) | 12 (5.5) | 2 (11.8) | 8 (36.4) |
| Missing, n | 8 | 13 | 3 | 6 | 4 | 189 | 7 | - |
|  |  |  |  |  |  |  |  |  |
| Physical activity |  |  |  |  |  |  |  |  |
| No or < 2 hours a week, n (%) | 189 (18.5) | 218 (18.1) | 131 (13.0) | 166 (13.2) | - | 2 (0.9) | 2 (11.8) | 3 (13.6) |
| 2-3 hours a week, n (%) | 389 (38.1) | 524 (43,5) | 429 (42.7) | 509 (40.6) | 8 (40.0) | 12 (5.5) | 4 (23.5) | 8 (36.4) |
| >3 hours a week, n (%) | 433 (42.4) | 449 (37.2) | 440 (43.8) | 569 (45.3) | 8 (40.0) | 13 (6.0) | 4 (23.5) | 11 (50.0) |
| Missing, n | 10 | 15 | 5 | 11 | 4 | 190 | 7 | - |
|  |  |  |  |  |  |  |  |  |
| Appearance and body functioning |  |  |  |  |  |  |  |  |
| Very satisfied, n (%) | 151 (14.8) | 203 (16.8) | 167 (16.6) | 235 (18.7) | - | 4 (1.8) | 3 (17.7) | 4 (18.2) |
| Medium satisfied, n (%) | 460 (45.1) | 586 (48.6) | 505 (50.3) | 671 (53.5) | 5 (25.0) | 16 (7.4) | 3 (17.7) | 13 (59.1) |
| Little satisfied, n (%) | 224 (21.9) | 257 (21.3) | 205 (20.4) | 197 (15.7) | 10 (50.0) | 5 (2.3) | 1 (5.9) | 3 (13.6) |
| Not satisfied at all, n (%) | 170 (16.7) | 135 (11.2) | 116 (11.5) | 71 (5.7) | 1 (5.0) | 2 (0.92) | 3 (17.7) | 2 (9.1) |
| Missing, n | 16 | 25 | 12 | 81 | 4 | 190 | 7 | - |
|  |  |  |  |  |  |  |  |  |
| Relapse, n (%) | 105 (10.3) | 77 (6.4) | 38 (3.8) | - | 3 (15.0) | 3 (1.4) | 1 (5.9) | - |
| Pain, n (%) | 330 (32.3) | 295 (24.5) | 282 (28.1) | 300 (23.9) | 7 (35.0) | 1 (0.5) | 3 (17.7) | 2 (9.1) |
| Fatigue, n (%) | 435 (42.6) | 287 (32.1) | 345 (34.3) | 111 (8.8) | 7 (35.0) | 4 (1.8) | 4 (23.5) | - |
| Lymphedema, n (%) | 163 (16.0) | 154 (12.8) | 141 (14.0) | 14 (1.1) | 1 (5.0) | 1 (0.5) | 2 (11.8) | - |
|  |  |  |  |  |  |  |  |  |
| Mobility, mean (SD) | 1.5 (0.8) | 1.5 (0.8) | 1.4 (0.8) | 1.3 (0.7) | 1.6 (0.6) | 1.5 (1.2) | 2.2 (1.5) | 1.2 (0.5) |
| Missing, n |  |  |  |  | 15 | 211 | 8 | 2 |
|  |  |  |  |  |  |  |  |  |
| Self-care, mean (SD) | 1.1 (0.4) | 1.1 (0.3) | 1.1 (0.3) | 1.1 (0.3) | 1.2 (0.5) | 1.0 (0.0) | 1.2 (0.4) | 1.0 (0.0) |
| Missing, n |  |  |  |  | 15 | 210 | 8 | 1 |
|  |  |  |  |  |  |  |  |  |
| Usual activities, mean (SD) | 1.7 (1.0) | 1.5 (0.8) | 1.6 (0.8) | 1.3 (0.6) | 2.5 (1.3) | 1.7 (1.2) | 2.0 (1.2) | 1.1 (0.2) |
| Missing, n |  |  |  |  | 16 | 211 | 8 | 1 |
|  |  |  |  |  |  |  |  |  |
| Pain/discomfort, mean (SD) | 2.2 (1.0) | 2.0 (0.8) | 2.1 (0.8) | 2.0 (0.8) | 1.7 (0.6) | 1.6 (0.9) | 2.8 (1.2) | 1.8 (0.8) |
| Missing, n |  |  |  |  | 17 | 212 | 8 | 1 |
|  |  |  |  |  |  |  |  |  |
| Anxiety/depression, mean (SD) | 1.7 (0.8) | 1.5 (0.8) | 1.5 (0.7) | 1.4 (0.6) | 1.7 (0.6) | 1.4 (0.9) | 1.8 (0.8) | 1.5 (0.8) |
| Missing, n |  |  |  |  | 17 | 212 | 8 | 8 |
|  |  |  |  |  |  |  |  |  |
| Health utility value, mean (SD) | 0.77 (0.16) | 0.81 (0.14) | 0.80 (0.13) | 0.83 (0.13) | - | - | - |  |
|  |  |  |  |  |  |  |  |  |
| Quality of life |  |  |  |  |  |  |  |  |
| Median (IQR), 0-100 | 60 (49-80) | 70 (50-80) | 70 (50-80) | 80 (70-90) |  |  |  |  |

^*^ p<0.05 for t-test comparing women with screen-detected cancer included and not included in the analyses

Abbreviations: SD – standard deviation, IQR – interquartile range

Figure A1:

A. Health utility values obtained from EQ-5D-5L for women with symptomatic, screen-detected and interval cancer over the time period from the third to the 14^th^ years since diagnosis, adjusted for stage, body mass index, education, physical activity, appearance and body functioning, relapse, age, fatigue, lymphedema, general pain and treatment received. Whiskers are standard errors.

B. Health utility values obtained from EQ-5D-5L among women with symptomatic, screen-detected and interval cancer, and women without breast cancer by age (years), adjusted for body mass index, education, physical activity, body image and functioning, age, fatigue, lymphedema, and general pain. Whiskers are standard errors.


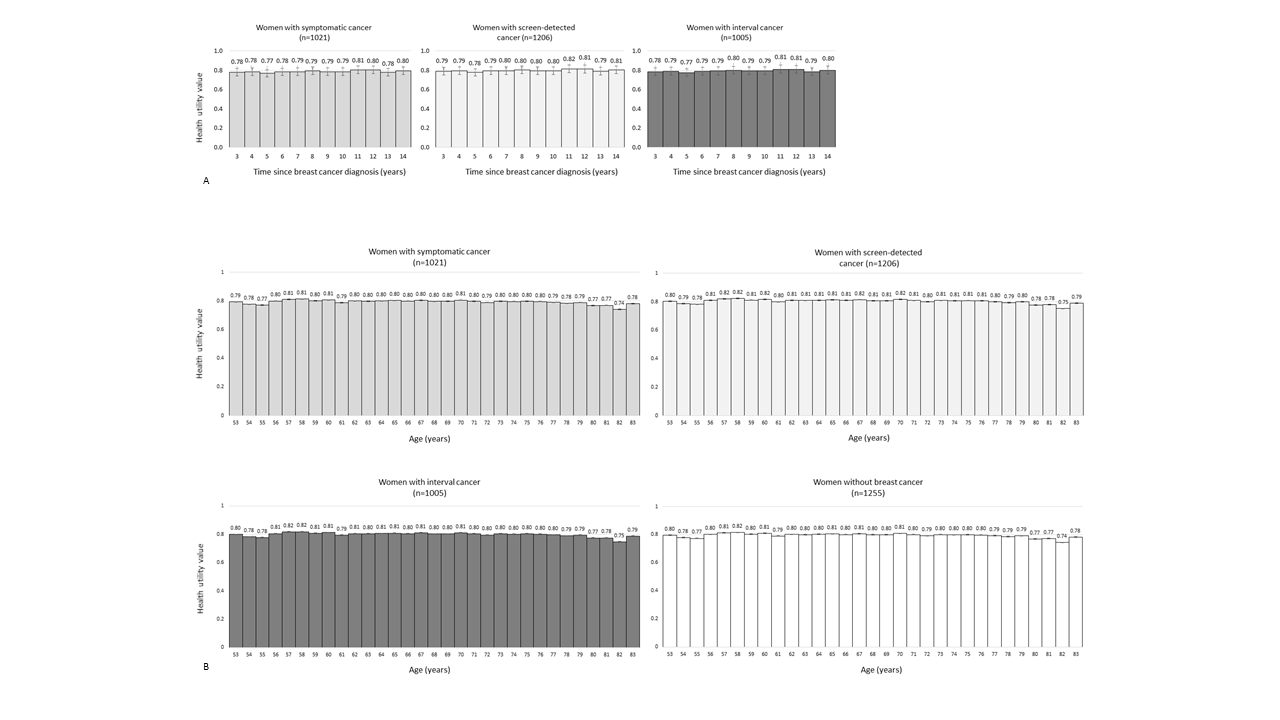

Supplement: Supplementary file 1 — Supplementary file1 (DOCX 421 KB) [file 11136_2021_3017_MOESM1_ESM.docx]
